# From Speech Semantics to Brain Activity—Timescales Are Key in Their Information Transfer

**DOI:** 10.1002/hbm.70379

**Published:** 2025-10-25

**Authors:** Saket Kumar, Philipp Klar, Yasir Çatal, Han‐Jen Chang, Friedemann Pulvermüller, Georg Northoff

**Affiliations:** ^1^ Brain and Mind Research Institute, Centre for Neural Dynamics, Faculty of Medicine The Royal's Institute of Mental Health Research & University of Ottawa, University of Ottawa Ottawa Ontario Canada; ^2^ Faculty of Mathematics and Natural Sciences, Institute of Experimental Psychology Heinrich Heine University of Düsseldorf Düsseldorf Germany; ^3^ Institute of Neuroscience and Medicine, Brain & Behaviour (INM‐7) Research Centre Jülich Jülich Germany; ^4^ Brain Language Laboratory, Department of Philosophy and Humanities Freie Universität Berlin Berlin Germany; ^5^ Cluster of Excellence ‘Matters of Activity. Image Space Material’ Humboldt Universität zu Berlin Berlin Germany; ^6^ Institute of Mental Health Research Royal Ottawa Hospital, University of Ottawa Ottawa Ontario Canada

**Keywords:** information transfer, semantics, speech, timescales

## Abstract

Fluctuating timescales are present in nature and are commonly observed in music, movies, brain activity, and speech. In human speech, semantic timescales span from single words to complete sentences and vary throughout conversation. Similarly, the brain's intrinsic neuronal timescales (INT), reflected in temporally correlated activity, carry information across time. How are these semantic and neuronal timescales related? Our combined semantic input and functional magnetic resonance imaging (fMRI) study using the 7 Tesla Human Connectome Project movie‐watching dataset reveals information transfer from speech's semantic timescales to the brain's INT. We extracted two semantic time‐series, sentence similarity and word depth, using Sentence‐BERT (SBERT) and WordNet, respectively. The timescales of both semantic signals and the brain's activity were quantified using the autocorrelation window (ACW), with a dynamic, time‐varying analysis approach. This allows testing for information transfer from the simultaneously varying semantic timescales to the brain's varying timescales via Transfer Entropy (TE). We report three main findings: (1) Sentence similarity and word depth time‐series exhibit high and systematic fluctuations over time. (2) Dynamic ACW analysis captures the dominant timescales in both semantic input (sentence similarity and word depth) and the brain's continuously varying INT. (3) Significant TE from the varying semantic timescales to the brain's simultaneously varying INT. We also demonstrate that the information transfer only emerges on the level of timescales, and is absent when comparing the two raw semantic input time‐series with the BOLD signal, respectively. Conclusively, we demonstrate the key role of timescales in the information transfer from semantic inputs to the brain's neural activity.

## Graphical abstract

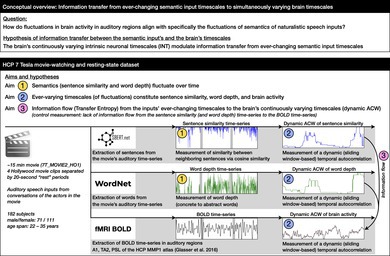

**Summary**

- Semantics of words and sentences have similar fluctuations in timescales.
- Information flows unidirectionally from semantics to brain activity in movie watching.
- Transfer Entropy is significant at the level of timescales, not at raw fluctuations.

## 1 Introduction

### 1.1 Fluctuating Timescales Underlying Human Speech Semantics and Brain Activity

The semantics of human speech, that is, the meaning of individual words and complete sentences, fluctuates continuously over the course of a conversation. For example, the semantic richness of words ranges from highly concrete (e.g., “dog”) to abstract concepts (e.g., “consciousness”), and can shift rapidly during speech. Naturally, complex human verbal communication goes beyond isolated words and relies on full sentences, which also display continuous variation throughout a conversation, such as in the semantic similarity between adjacent sentences. From a dynamic perspective, speech unfolds across multiple timescales, ranging from shorter timescales for individual words to longer ones for complete sentences. Crucially, these semantic timescales do not fluctuate randomly. Just as the temporal structure of words and sentences contributes to the meaning of a message, their underlying temporal dynamics also exhibit non‐random patterns.

These dynamics show significant temporal autocorrelation, characterized by specific and recurring temporal patterns that convey meaningful information, as demonstrated by studies that recorded and analyzed human speech (Sacks et al. 1974; Voss and Clarke 1975; Sabanal and Nakagawa 1996; Luque et al. 2015; Menninghaus et al. 2018). Temporal autocorrelation refers to the correlation of a signal with a time‐lagged version of itself, providing a measure of the non‐random temporal structure or patterns that carry information within the signal. The signal's autocorrelation thus reflects the degree to which earlier data points in a time‐series positively or negatively correlate with later ones. In this way, temporal autocorrelation quantifies how much “memory” a signal carries, namely, the transmission or integration of information over time (Williams 1997). The autocorrelation window (ACW) defines the specific time lag used to compute this statistic, representing the duration over which the signal retains meaningful autocorrelation before falling below a set threshold. Commonly used metrics include ACW‐50, the point at which the autocorrelation function first drops below *r* = 0.5, and ACW‐0, the first zero crossing of the autocorrelation function (*r* = 0) (Bassingthwaighte et al., 1994; Williams 1997; Golesorkhi et al. 2021; Honey et al. 2012).

Timescales that exhibit significant degrees of autocorrelation, or memory over time, also underlie human brain activity. In electrophysiological recordings such as electroencephalography (EEG) and magnetoencephalography (MEG), as well as in functional magnetic resonance imaging (fMRI), these temporally structured patterns are often referred to as intrinsic neuronal timescales (INT) (Wolff et al. 2022; Golesorkhi et al. 2021; Hasson et al. 2015; Yeshurun et al. 2021) and are frequently measured using the signal‘s ACW (Sabanal and Nakagawa 1996; Smith et al. 2022; Luque et al. 2015; Huang et al. 2018; Watanabe et al. 2019; Golesorkhi et al. 2021; Wolff et al. 2022; Klar et al. 2023). EEG and fMRI studies have demonstrated that INT are involved in the processing of environmental stimuli, such as auditory inputs during movie‐watching (Lerner et al. 2011; Jääskeläinen et al. 2021; Hasson et al. 2015; Cavanagh et al. 2020; Zilio et al. 2020; Zeraati et al. 2023; Northoff et al. 2023; Golesorkhi et al. 2021; Klar et al. 2023). During auditory processing, lower‐order sensory regions exhibit relatively short timescales, as indicated by ACW values typically within a few seconds in fMRI (Lerner et al. 2011; Hasson et al. 2015; Ito et al. 2020). In contrast, higher‐order association areas are characterized by much longer timescales, reflected in ACW values that extend over several seconds, indicating a greater capacity for temporal integration of information over time, such as during the processing of complete sentences (Yeshurun et al. 2021; Raut et al. 2020; Hasson et al. 2015; Golesorkhi et al. 2021; Wolff et al. 2022; Wolman et al. 2023). While these findings provide strong evidence that the brain ‘s INT support successful speech processing, our understanding of the brain's semantic information processing remains yet incomplete. Notably, the presence of information‐carrying timescales characterized by high autocorrelation in both speech semantics and the BOLD signals raises the question of whether the dynamics of these two processes are functionally related through their timescales. Our combined fMRI and speech input study seeks to address this question and to overcome the three gaps, which we will elaborate on subsequently.

We identified two key methodological gaps in the current literature. First, although previous neuroimaging studies have analysed datasets using human speech as input (e.g., Wolff et al. 2022; Klar et al. 2023), they have not examined the inherent timescales of speech semantics themselves, nor how these semantic timescales are processed by and related to the corresponding timescales in the BOLD signals. For example, the well‐known work by Hasson and colleagues investigates temporal receptive windows (TRWs): the refer to the duration of time before a response where the received sensory information may affect the response (Hasson et al. 2008). The TRWs are often investigated by using inter‐subject correlation (ISC), but this approach is relatively static, relying on averaged metrics across entire time‐series (Lerner et al. 2011; Hasson et al. 2015; Ito et al. 2020)—this misses the continuous change and thus dynamics inherent in our speech semantics. Furthermore, ISC only provides a relatively indirect measure of stimulus processing, as it does not capture the actual temporal dynamics of the input, for example, the semantic fluctuations by themselves, the “ground truth” necessary for a direct comparison between brain activity and input dynamics.

Second, the semantics of speech and the BOLD fluctuations are not static processes; as outlined above, they exhibit significant fluctuations in their timescales over time. A static approach, where the ACW is computed once across the entire recording, would average out these meaningful temporal structures that reveal specific patterns of information or memory in both semantics and brain activity (see Golesorkhi et al. 2021; Wolff et al. 2022; Wolman et al. 2023 for studies linking timescales to brain function and information transfer). Recent work from our group (Klar et al. 2023) employed a dynamic, sliding window‐based approach and demonstrated that both the brain's spectral exponent (measured by linear regression of the power spectrum on a log–log plot of frequency versus power reflecting the dynamics of the power law exponent's values over time) and dynamic ISC display non‐random temporal patterns during a movie‐watching paradigm in visual and auditory brain regions. Therefore, to capture the evolving temporal structure of the timescales in both speech semantics and the BOLD signals, a dynamic approach, such as a sliding window analysis (Barttfeld et al. 2015; Hudetz et al. 2015; Tagliazucchi and Laufs 2014; Laumann et al. 2017; Huang et al. 2018), is necessary, rather than the commonly used static, run‐based averaging methods of INT (Lerner et al. 2011; Hasson et al. 2015; Ito et al. 2020; Wolff et al. 2022; Klar et al. 2023).

### 1.2 Is There Information Transfer From Semantic Speech to Neuronal Timescales?

The primary question our combined fMRI and semantic input study seeks to address is whether the continuously varying timescales of speech semantics, measured through a dynamic (sliding window‐based) ACW analysis of the movie's word depth (using WordNet; Miller 1995; Oram 2001) and sentence similarity (using Sentence‐BERT (SBERT); Reimers and Gurevych 2019), are systematically processed by the brain's own fluctuating INT. BERT stands for “Bidirectional Encoder Representations from Transformers”. BERT is a language model developed by Google and published in 2018 (Devlin et al. 2019). SBERT is an extension of BERT that uses siamese and triplet network architectures to derive semantically meaningful sentence embeddings, which can be compared using cosine similarity (Reimers and Gurevych 2019). Cosine similarity measures the similarity between two sets of information, such as consecutive sentences, by calculating the cosine of the angle between their respective vectors. A cosine similarity value closer to one means that two sentences are more similar. Pursuing a dynamic approach, we constructed a sentence similarity time‐series via SBERT and then calculate the cosine similarity between adjacent sentences. Next, we build a word depth time‐series based on the movie's auditory data using WordNet (Miller 1995; Oram 2001). WordNet is a lexical database of English that groups nouns, verbs, adjectives, and adverbs into sets of cognitive synonyms, known as synsets. WordNet assigns each word a depth value that reflects the word's level of abstraction, ranging from concrete to abstract concepts. For example, the word “pineapple” has a lower depth than the more abstract term “food”, which is broader in meaning. WordNet assigns a lower depth value to more concrete words and a higher depth value to abstract words (Miller 1995; Oram 2001). (A detailed explanation of our SBERT and WordNet analyses can be found in the methods section).

We ask whether the brain's processing of these semantic timescales at the word and sentence levels enables a significant semantic input‐to‐brain information transfer. This transfer is quantified using Transfer Entropy (TE; Schreiber 2000; Ikegwu et al. 2020), applied to the sliding window‐based ACW analysis between word‐level timescales and timescales of BOLD fluctuations, as well as between sentence‐level timescales and timescales of BOLD fluctuations. TE has been used in previous neuroimaging studies (for reviews, see Barnett et al. 2009; Seth et al. 2015). In brief, TE captures the mutual information between past values of one process (such as the semantic dynamic ACW) and present values of another process (such as the brain's dynamic ACW) (Schreiber 2000; Bossomaier et al. 2018). TE conditions (or controls for) information transfer from past to present brain timescales, thus focusing the analysis on the information transfer exclusively from the semantic timescales to the brain's timescales or INT, irrespective of the brain's previous timescales (see the methods section for details on TE).

We chose word depth and sentence similarity for several reasons. First, WordNet and SBERT are well‐established methods for analyzing human speech semantics and have demonstrated reliable, replicable results across experiments (see, e.g., Budanitsky and Hirst 2006; Wang and Kuo 2020; Yan et al. 2021). Second, word depth and sentence similarity capture complementary semantic features operating at different timescales: word depth reflects context‐independent meaning at shorter timescales, whereas sentence similarity captures context‐dependent meaning where the combined semantic content of a sentence exceeds the sum of its individual words—this requires the brain to integrate distinct word inputs over longer timescales to understand the meaning of a full sentence (Lerner et al. 2011; Hasson et al. 2015; Wolff et al. 2022). From a neuroscientific perspective on semantic processing, it is particularly interesting to determine whether significant information transfer from semantic speech input to brain activity occurs already at the level of individual words or whether such transfer is limited to the sentence level. Even more important is the question of whether such information transfer in both words and sentences is mediated by their differential timescales, for example, shorter and longer.

To achieve this task, we analysed the 7 Tesla movie‐watching dataset from the Human Connectome Project (HCP) and selected the MOVIE2\_HO1 run, which is particularly well‐suited to our aims. This run consists of five Hollywood movie clips featuring continuous dialogues between actors. In contrast, the other three runs from the 7 Tesla HCP movie‐watching dataset (MOVIE1\_CC1, MOVIE3\_CC2, and MOVIE4\_HO2) either contained too many scenes without ongoing speech, such as action sequences or primarily featured non‐verbal scenes, such as nature footage. After extracting the auditory time‐series from the movie clips, we constructed a sentence similarity time‐series using SBERT (Reimers and Gurevych 2019) and a word depth time‐series using WordNet (Miller 1995; Oram 2001) (see the aims and methods section for details on the two semantic analyses). Note that neither SBERT nor WordNet captures the full spectrum of semantic content, particularly more nuanced dimensions such as semantic priming, surprise, or meaning in a multi‐dimensional semantic space. For a detailed discussion, see the limitations section.

In the next step, we applied a dynamic (sliding window‐based) ACW analysis to examine the fluctuating timescales in the word depth and sentence similarity time‐series, as well as in the brain's blood‐oxygen‐level‐dependent (BOLD) signal. This analysis focused on three auditory regions defined in the HCP MMP 1.0 atlas (Glasser et al. 2016): the primary auditory cortex (A1), region TA2 located on the temporal lobe, and region PSL (Peri‐Sylvian Language area, associated with Wernicke's region), which lies at the temporo‐parieto‐occipital junction and is anatomically even more distant from the brain's primary sensory inputs than region TA2. In addition, we conducted a whole‐brain analysis of information transfer, assessing TE from semantic input to brain timescales across all 360 regions of the HCP MMP atlas to generate a comprehensive whole‐brain TE map.

### 1.3 Overview of Specific Aims

Aim One: We first aim to demonstrate that both sentence similarity and word depth fluctuate continuously over time in a systematic way. To achieve this, we construct a sentence similarity time‐series using Sentence‐BERT (SBERT) (Reimers and Gurevych 2019) and a word depth time series using WordNet (Miller 1995; Oram 2001), both based on auditory data from the movie.

Aim Two: Our second objective is to show that both sentence similarity and word depth time‐series, as well as the brain's BOLD signal, all exhibit varying degrees of memory or information over time. This is measured using our dynamic ACW analysis, which should reveal significant temporal fluctuations during the movie run. Conversely, we expect the brain's dynamic ACW to show significantly lower variance during resting‐state conditions, due to the absence of continuous semantic variation present during movie‐viewing.

Aim Three: Finally, to directly link semantic and brain timescales, we use TE (Barnett et al. 2009; Seth et al. 2015) to analyse the direction of information flow from the dynamic ACW of sentence similarity and word depth to the brain's dynamic ACW. We hypothesize that significant TE will be observed only at the level of these continuously varying ACW timescales. In contrast, we expect no significant information transfer (non‐significant TE) between the raw sentence similarity and word depth time‐series and the brain's BOLD signal on the other.

Note that we also validated our TE analysis through several supplementary control measurements (see the Supporting Information: Analyses section for details). First, we assessed TE from the sentence similarity and word depth time series to the brain's BOLD time series, where we expected to find non‐significant TE. Second, we calculated TE in the reverse direction—from the brain's BOLD time series to the semantic measures, anticipating non‐significant information flow at both the level of the dynamic ACW and the raw time series. Third, we applied Markov Block Bootstrapping, a block‐based shuffling method, to both sentence similarity and word depth time series and then calculated TE from these shuffled inputs to the brain. We expected that disrupting the temporal structure of the semantic inputs would abolish information flow to the brain, resulting in non‐significant TE.

**FIGURE 1:**
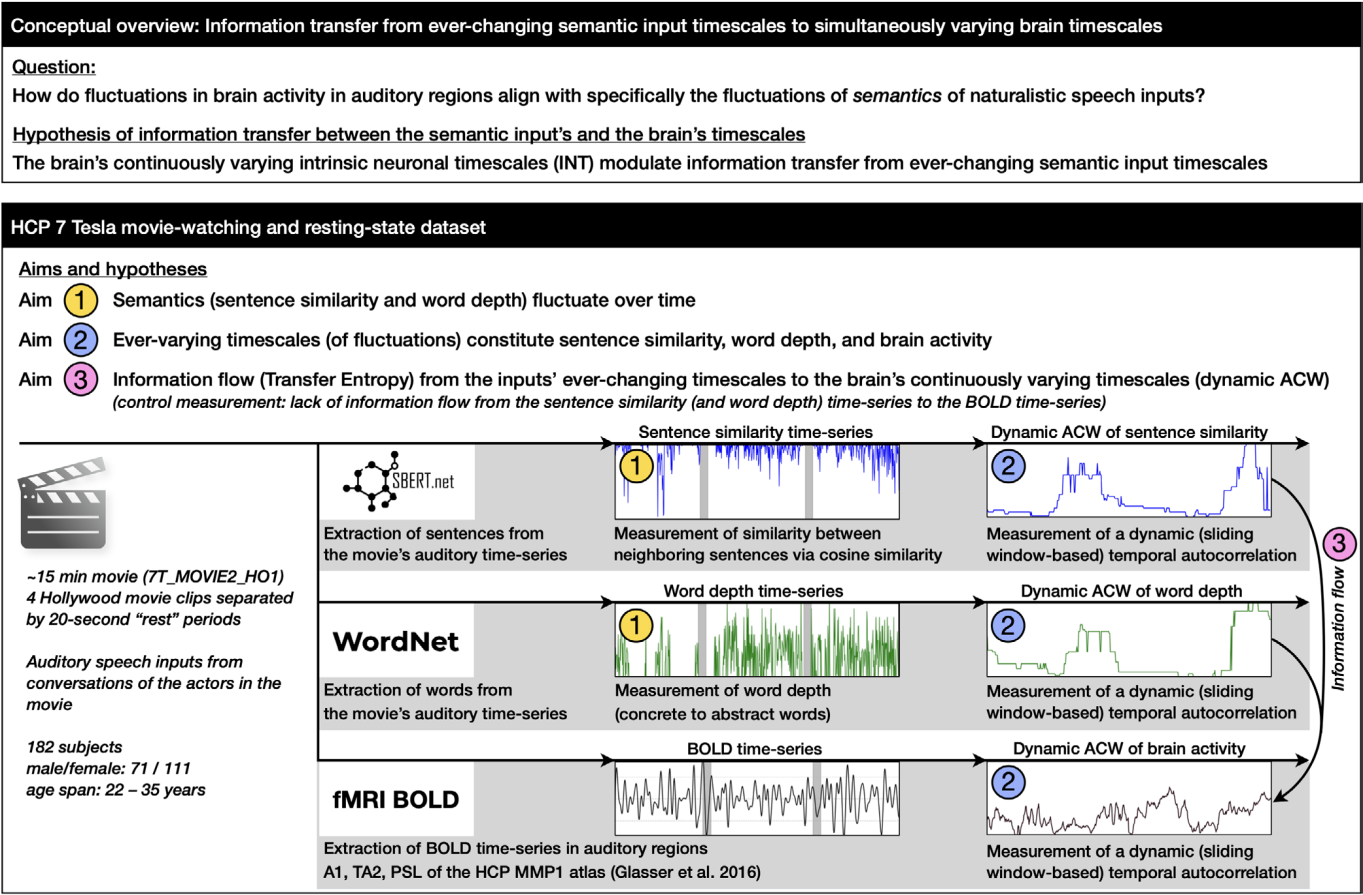
Overview. We analyzed sentence similarity (SBERT) and word depth (WordNet) from the HCP 7 T MOVIE2_HO1.mp4 using dynamic ACW analysis. Brain activity in A1, TA2, and PSL regions was similarly analyzed. Transfer Entropy measured the information flow between semantic and brain timescales.

In summary, our combined fMRI and semantic input study extends previous neuroimaging research on INT (Lerner et al. 2011; Hasson et al. 2015; Ito et al. 2020; Jääskeläinen et al. 2021; Cavanagh et al. 2020; Zilio et al. 2020; Zeraati et al. 2023; Northoff et al. 2023; Golesorkhi et al. 2021) by directly demonstrating the flow of information from continuously changing semantic timescales to the brain's simultaneously fluctuating INT, suggesting this interaction as a potential mechanism supporting speech comprehension. Figure 1 provides a conceptual overview of our study.

## 2 Methods

### 2.1 Subjects and Study Design

We selected 182 subjects (male/female: 71/111; age span: 22–35 years) from the preprocessed young adults Human Connectome Project (HCP, db.humanconnectome.org/data/projects/HCP_1200/) and ICA‐FIX denoised (Salimi‐Khorshidi et al. 2014; Glasser et al. 2013; Griffanti et al. 2014) 7 Tesla fMRI dataset. The scanning protocol, participant recruitment procedure, informed written consent forms, and consent to share de‐identified data were approved by the Washington University institutional review board (Van Essen et al. 2013). We selected the so‐called 7T_REST1 (resting‐state) and 7T_MOVIE2_HO1 (movie‐watching) runs, both recorded in the posterior‐to‐anterior (PA) phase encoding direction. During the resting‐state, subjects had to keep their eyes open while fixating a projected bright cross‐hair on a dark background presented in a darkened room. During the movie‐watching run, subjects watched four Hollywood movie clips of the following lengths. Three 20‐s rest periods separate the four movie clips. The movie‐watching run included two additional 20‐s rest periods before the first and after the last clip.

### 2.2 Data Acquisition

The dataset was recorded with Siemens MAGNETOM 7 Tesla MRI scanner housed at the Center for Magnetic Resonance (CMRR) at the University of Minnesota in Minneapolis that acquired whole‐brain scans. Physiological cardiac and respiratory recordings were not acquired. The scanner acquired gradient‐echo echo‐planar imaging (EPI) recordings with the following imaging parameters: time repetition = 1000 ms, time echo = 22.2 ms, flip angle = 45°, slice thickness = 1.6 mm (85 slices, 1.6 mm isotropic voxels for functional runs), field of view = 208 × 208 mm (RO × PE), matrix = 130 × 130 (RO × PE), multiband factor = 5, echo spacing = 0.64 ms, image acceleration factor = 2, partial Fourier sampling = 7/8, BW = 1924 Hz/Px. The complete scanning protocol is available at https://www.humanconnectome.org/hcp‐protocols‐ya‐7t‐imaging.

### 2.3 Preprocessing

We investigated the minimally preprocessed (Glasser et al. 2013) and ICA‐FIX denoised (Salimi‐Khorshidi et al. 2014; Griffanti et al. 2014) 7 Tesla HCP dataset in standard volume NIfTI space. A comprehensive preprocessing description is available in Glasser et al. (2013). Briefly, HCP preprocessing and ICA‐FIX denoising included the following steps: (1) removal of spatial artifacts and distortions, such as correction of MR gradient‐nonlinearity‐induced distortions for the anatomical scans; (2) nonlinear spatial normalization of the anatomical scans to MNI152 space and subsequent nonlinear functional to anatomical alignment (normalization) with a single spline interpolation that minimizes interpolation‐induced blurring; (3) realignment of functional scans to compensate for subject head motion with a 6 DOF FLIRT registration of each frame to the single‐bad reference image; (4) reduction of bias field; (5) normalization of the functional scans to a global mean and masking of the data with a final brain mask; and (6) cleaning of structured noise (denoising) via a pair of independent component analyses (MELODIC) with the FSL tool FIX to remove artefactual components. The combination of ICA with the automated component classifier FIX was specifically trained on the HCP data. The HCP preprocessing pipeline excluded spatial smoothing, temporal filtering, slice timing correction, and motion censoring. We removed the first and the last 20‐s rest periods of the movie run. We also applied a linear detrending on the BOLD time‐series to remove some linear trends left over in the preprocessed HCP dataset.

### 2.4 Dynamic ACW Analysis of the Brain's BOLD Signal

The autocorrelation function is a dimensionless statistic (Williams 1997). It measures repeating temporal patterns in time‐series data. Earlier time points can positively or negatively correlate with later values. Autocorrelation measurements find application in neuroimaging to estimate the length of intrinsic neuronal timescales (Wolff et al. 2022; Cavanagh et al. 2020; Wolman et al. 2023). Higher compared to lower autocorrelation values can represent memory, the transmission or integration of information over time, and may reflect the processing of internal and external inputs. The autocorrelation window zero refers to the chosen time lag for computing the autocorrelation statistic, that is, where ACW describes the window length of the autocorrelation function before the signal's autocorrelation first reaches or drops below zero (Klar et al. 2023; Watanabe et al. 2019; Williams 1997). Besides ACW‐0, there is also ACW‐50, where the ACF reaches to a value of 0.5, and ACW‐e^‐1, where ACF reaches 0.37. However, ACW‐0 has known to be better able to distinguish between core and periphery regions (Golesorkhi et al. 2021) as well as in differentiating between self specific and non‐self specific activities (Northoff et al. 2023). We applied a sliding window analysis previously applied in fMRI studies (Barttfeld et al. 2015; Hudetz et al. 2015; Tagliazucchi and Laufs 2014; Laumann et al. 2017; Huang et al. 2018). We used a 60‐s window with a one‐second step increase (equal to one fMRI time repetition or TR). Before computing the ACW we applied a third‐order forward‐backward (effectively a sixth‐order) Butterworth filter bandpass filter in the 0.05–0.5 Hz frequency band on the complete time‐series. The lower frequency (0.05 Hz) was chosen to include at least three cycles of the slowest frequency within the 60‐s window. The upper frequency (0.5 Hz) represents the Nyquist frequency due to the dataset's 1 Hz sampling rate. We decided to go up to the Nyquist frequency instead of constraining the frequency band to 0.1 Hz because empirical evidence suggests that frequencies beyond the commonly chosen upper limit of 0.1 Hz can contain meaningful information (Gohel and Biswal 2015; Shirer et al. 2015; Caballero‐Gaudes and Reynolds 2017). We analyzed the blood‐oxygen‐level‐dependent (BOLD) ACW for each window based on the region‐based mean time‐series per subject. Finally, we took the mean dynamic ACW across subjects per region. The autocorrelation formula (Equation 1) at a given lag contains two ingredients, namely autocovariance (Equation 2) and variance (Equation 3). In these formulas, *N* is the number of sampling points, t is a time point in the time‐series, m is the lag, and *x* is the mean of the entire time‐series (a constant). The autocorrelation for a specific lag is the autocovariance for that lag as standardized by the variance of the observations, namely the autocovariance divided by the variance (Williams 1997).

(1) $$\text{autocorrelation}=\frac{\text{autocovariance}}{\text{variance}}$$

(2) $$\text{autocovariance}=\frac{1}{N}{\sum_{t=1}}^{N-m}\left(x_{t}-\bar{x}\right)\left(x_{t+m}-\bar{x}\right)$$

(3) $$\text{variance}=\frac{1}{N}{\sum_{t=1}}^{N}{\left(x_{t}-\bar{x}\right)}^{2}$$

### 2.5 Dynamic ACW Analysis of the Movie's Semantic Inputs (SBERT and WordNet)

We subsequently explain how we investigated the speech semantics of the 7T HCP movie file. We first extracted the auditory time‐series from the 7T_MOVIE2_HO1.mp4 file provided in the Human Connectome Project dataset. We then converted the audio from stereo to mono. Next, we used the Google Cloud Speech‐to‐Text API (https://cloud.google.com/speech‐to‐text) to extract spoken words from the movie into text. We also extracted the start and end times of each word. Finally, we manually checked for transcription errors to ensure optimal quality of the word extraction. We applied fixes where necessary. These fixes include: (1) misrecognition of words (e.g., “fault” instead of “vault”). (2) Incorrect start or end times. (3) Failure to recognize certain words or phrases. We applied the same sliding window approach with a 60‐s window with a one‐second step increase for the movie semantics as applied to the brain's BOLD signal. We concatenated all words into one segment for each window. We also included words if their starting time fell within our 60‐s window, ensuring the inclusion of boundary words. We saved each segment with the window's starting and end time, creating a time‐series of sentences representing the dialogue between the movie's actors. The creation of such a speech time‐series then allowed us to use two natural language processing metrics to extract semantics: one based on sentence similarity, namely Sentence‐BERT (SBERT) (Reimers and Gurevych 2019), and the other on word depth, namely WordNet (Miller 1995; Oram 2001).

Sentence‐BERT (SBERT): BERT stands for bidirectional encoder representations from transformers. BERT is a language model by Google. It was introduced in 2018 (Devlin et al. 2018). SBERT is a modification of BERT that uses siamese and triplet network structures. It can derive semantically meaningful sentence embeddings. These are comparable by using cosine similarity (Reimers and Gurevych 2019). Cosine similarity measures the similarity between sets of information, such as the similarity between two consecutive sentences. In our study, cosine similarity measures the cosine of the angle between vectors (two sentences) and determines if the vectors point in the same or similar direction (angle). The closer cosine similarity is to one, the more similar the sentences. Therefore, cosine similarity ignores measuring the magnitude similarity of vectors; instead, it compares the cosine of the angle between vectors or the similarity of semantics. Using SBERT, we construct a semantic input time‐series via cosine similarity and subsequently apply our dynamic ACW analysis to this semantic SBERT time‐series.

WordNet: The second method uses WordNet (Miller 1995; Oram 2001). We applied WordNet to construct a semantic input time‐series. WordNet is a lexical database of English that includes nouns, verbs, adjectives, and adverbs grouped into sets of cognitive synonyms. Here, each word is assigned a specific word depth from concrete to more abstract words, such as from “pineapple” over “fruit” to “food.” WordNet attributes a higher word depth to concrete than abstract words (Miller 1995; Oram 2001). WordNet focuses on discrete words, unlike the cosine similarity in the SBERT method (which spans across words to analyse sentences). Words were binned into one‐second intervals without overlap, matching the sampling rate of the fMRI BOLD signal. We calculated the average word depth for each bin based on WordNet's semantic hierarchy, which assigns depth values to words. We assigned a word depth of zero to bins that lacked words. We finally apply our dynamic ACW analysis on the semantic WordNet time‐series.

### 2.6 Transfer Entropy (TE) Between the Semantic Input's and the Brain's Dynamic ACW

Transfer Entropy (TE) (Schreiber 2000; Ikegwu et al. 2020) is a measurement of information transfer previously applied in neuroimaging studies (for reviews, see Barnett et al. 2009; Seth et al. 2015). TE measures mutual information between past values of one process, such as the input's semantic dynamic ACW, and present values of another process, such as the brain's dynamic ACW (Schreiber 2000; Bossomaier et al. 2018). TE conditions or controls information transfer from past to present brain timescales, consequently allowing the analysis of information transfer exclusively from the input's semantics to the brain's INT irrespective of the brain's past timescales. Unlike mutual information, TE includes the application of lags between the input's and brain's timescales by application of time lags. That TE applies time lags between the input's semantic and the brain's dynamic ACW is appropriate given that information transfer is unidirectional from the input to the brain and not instantaneous due to the BOLD's sluggish nature (Logothetis 2008). We estimated the significance of TE by comparing with surrogate TE values calculated from shuffled datasets. We applied the Markov bootstrap procedure to shuffle the timeseries. This approach involves dividing each subject's BOLD time series into blocks with lengths corresponding to the average dynamic ACW timescale, as determined by a sliding window approach, which are then shuffled within each subject's time series. This procedure preserves local temporal order (Cerqueti et al. 2017) which ensures that the shuffled timeseries still carries mutual information with its past, unlike a random shuffling method which reduces any relevant timescales into white‐noise, making it completely unpredictable and unsuitable for making an inference. For each shuffled dataset, TE was recalculated between the dynamic ACW of the input and the brain. This procedure was repeated 1.000 times to produce a distribution of shuffled TE values. The *p* value for TE was then estimated as the proportion of shuffled TE values greater than the observed (non‐shuffled) TE, divided by 1000.

### 2.7 Regions of Interest (ROIs)

We assessed three auditory input regions of the HCP MMP 1.0 “Glasser 360” atlas (Glasser et al. 2016). The atlas used multi‐modal magnetic resonance images and specified 180 areas per hemisphere based on cortical architecture, function, connectivity, and topography in a group average of 210 healthy young adults. (See “supporting information Neuroanatomical Results” for details of the regions in Glasser et al. 2016 available at https://www.nature.com/articles/nature18933). The three investigated regions span from lower‐order to higher‐order regions. They are A1 part of the early auditory regions, TA2 part of the auditory association regions, and PSL (Peri‐Sylvian Language area) part of the temporo‐parieto‐occipital junction. While the former area is essential for auditory processing, the latter two are part of the left perisylvian areas where substantial lesions regularly lead to aphasia (neurologically induced language impairment). We selected the early A1 region as the canonical input region of the auditory cortex (Glasser et al. 2016; Rolls et al. 2023). Next, we selected region TA2, located on the anterior temporal lobe between regions A1 and PSL. TA2 is anatomically and functionally located before the so‐called STS regions of the HCP MMP 1.0 atlas (Glasser et al. 2016; Rolls et al. 2023). The STS regions, in turn, receive mixed inputs from both auditory and visual regions. Finally, we included the Peri‐Sylvian Language area that forms part of cortical association areas (Rolls et al. 2022) which is both linked to TA2 and one of the areas of cortex known to both activate and functionally contribute to semantic processing (Binder and Desai, TiCS 2011; Pulvermuller, TiCS, 2013; Prog Neurobiol 2018). To investigate if a significant semantic input‐to‐brain information transfer extends beyond early auditory processing in auditory (A1) and auditory association (TA2) regions to the PSL that is well known to be directly implicated in language processing. Future studies may want to assess other regions like anterior temporal lobe and posterior superior temporal sulcus that are well known to be implicated in semantic processing (Pulvermüller 1999, 2018).

### 2.8 Inter‐Subject Correlation and Whole Brain Analysis

Beyond the three core ROIs, we assessed Inter‐Subject Correlation (ISC) across all 360 cortical regions of the Glasser atlas (Glasser et al. 2016). Since it is a big dataset, we extracted the BOLD timeseries of four regions of interest at a time using GNU parallel (Tange 2025). We bandpassed the BOLD timeseries of each ROI and then segmented into overlapping 60 s sliding windows. For each window, we calculated pair‐wise Pearson correlation and the median was taken as the ISC for the window. We averaged over all windows to get the ISC value for the ROI. We also calculated correlation matrix between all ROIs and then took the mean of all matrices as the average correlation. We grouped the ROIs into 22 functional networks according to the Neuroanatomical supporting information—results of Glasser et al. (2016) (https://pmc.ncbi.nlm.nih.gov/articles/instance/4990127/bin/NIHMS68870‐supplement‐Neuroanatomical_Supplementary_Results.pdf), and median correlation matrices were visualized as triangular plots. We repeated this analysis for the dynamic ACW timeseries of all the ROIs and presented the two triangular plots for comparison between the difference in ISC at the level of raw and dynamic ACW fluctuations.

To increase the generalization of our results and interpretations into studies involving semantics, we also extended our Transfer Entropy analysis to the whole brain. Using the same pre‐processing steps for both of the semantic input and the BOLD signals, we calculated TE from the Sentence Similarity and the Word Depth timeseries to the BOLD signals at the level of dynamic ACW for the first lag for all 360 regions of interest. We used nilearn (Abraham et al. 2014) to plot the brain surface maps of the left and right hemispheres.

### 2.9 Supplementary Analyses

#### 2.9.1 Supplementary Analysis 1: Power Spectral Density (PSD) of BOLD Time‐Series for Task and Rest States

To compare the frequency characteristics of brain activity across different regions during task and rest states, we computed the power spectral density (PSD) of the BOLD time series for the A1, TA2, and PSL regions. We removed the first and last 20 samples to mitigate edge effects, applied a linear detrending, and a bandpass filter (0.05–0.5 Hz) to isolate the relevant frequency components. The PSD was estimated using Welch's method (Welch 1967) and the profiles for task (movie run) and rest conditions were plotted for comparison.

#### 2.9.2 Supplementary Analysis 2: PSD of Semantic Timescales (Sentence Similarity and Word Depth)

To examine the frequency characteristics of semantic timescales, we analysed the PSD of sentence similarity and word depth data. After removing the first and last 20 samples to mitigate edge effects, PSDs were computed using Welch's method (Welch 1967), and plotted for comparison.

#### 2.9.3 Supplementary Analysis 3: Transfer Entropy From the BOLD Time‐Series to Semantics

Since TE can also be bidirectional, we decided to apply our analysis of Transfer Entropy to see if the BOLD time‐series can predict the two semantic time‐series or not. We expect to observe no significant TE either at the level of raw time‐series or at the level of dynamic ACW. Significance was calculated by Markov Block Bootstrapping of the BOLD time‐series.

#### 2.9.4 Supplementary Analysis 4: Transfer Entropy Using Shuffled Semantic Time‐Series

We also shuffled the sentence similarity and the word depth time‐series using Markov Block Bootstrapping with 1000 iterations, and calculated TE from the average of the shuffled versions of semantics to the BOLD time‐series, including the dynamic fluctuations of the ACW. We expect shuffling to alter the timescales of the semantics and no significant TE should be observed. Significance was calculated by Markov Block Bootstrapping of the BOLD time‐series.

#### 2.9.5 Supplementary Analysis 5: Transfer Entropy (Input to Brain) for Individual Movie Durations

We calculated Transfer Entropy from Sentence Similarity and Word Depth for the durations of each movie durations. These are (20, 246), (267, 525), (545, 794), and (815, 897). We find information flow for each movie suggesting that the movies are behaving in quasi‐independent independent manner within the whole 15 min movie.

#### 2.9.6 Supplementary Analysis 6: Transfer Entropy for Longer Window Sizes

We calculated Transfer Entropy from the dynamic ACW of the two semantic inputs (Sentence Similarity and Word Depth) to the dynamic ACW of the BOLD signals for the three ROIs (A1, TA2 and PSL) for window sizes 60, 120, 180, 240, 300, 360, 420, 480, and 540 s. We find TE to be inversely correlated with window sizes. It decreases and starts turning insignificant at very long windows.

## 3 Results

### 3.1 Semantic Dynamics—Fluctuations of Sentence Similarity and Word Depth

Our first aim focused on the semantics of words and sentences spoken by the actors throughout the movie. We constructed a sentence similarity time‐series using Sentence‐BERT (SBERT) (Reimers and Gurevych 2019) and a word depth time‐series using WordNet (Miller 1995; Oram 2001).

*Sentence similarity time‐series:* The cosine similarity between neighbouring sentences, used as a measure of sentence similarity, ranged from 0 to 1. Zero indicates no similarity and one indicates identical sentences. Generally, we observed high cosine similarity values. Most values fluctuated between approximately 0.94 and 1, suggesting that neighbouring sentences were similar.

*Word depth time‐series:* Word depth fluctuated continuously throughout the movie, with values ranging from 0 (no speech or words) to 7 (highly abstract words). Intermediate values (e.g., 3 and 5) corresponded to words that were neither too concrete nor too abstract.

**FIGURE 2:**
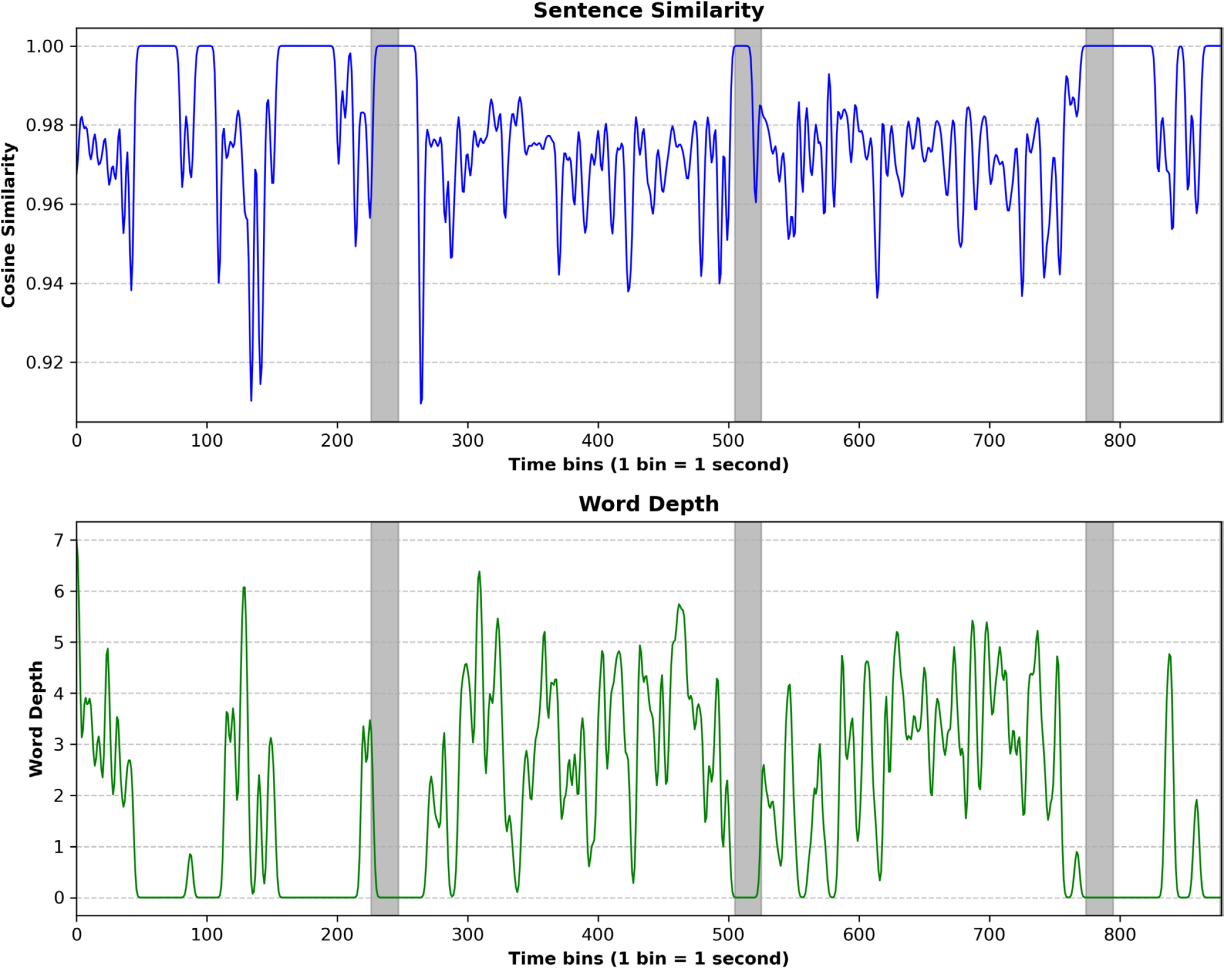
Semantic fluctuations. Sentence similarity time‐series (blue) constructed with Sentence‐BERT (SBERT) measured by cosine similarity and word depth time‐series (green) constructed with WordNet. The gray vertical bars represent the 20‐s rest periods between the movie's single clips.

Together, both the sentence similarity and word depth time‐series demonstrate that the semantics of human speech are dynamic, shown via the continuously varying sentence similarity and continuous changes from concrete to abstract words. Figure 2 displays the sentence similarity and word depth time‐series.

### 3.2 Semantic Timescales—Dynamic ACW of Sentence Similarity and Word Depth

After constructing the sentence similarity and word depth time‐series in aim one, we now investigate if the two semantic time‐series exhibit continuously changing timescales measured via our dynamic ACW approach.

*Dynamic ACW of sentence similarity:* We observed continuously varying ACW values for sentence similarity ranging from 1 to 20 s over the movie's course.

*Dynamic ACW of word depth:* We also observed continuously varying ACW values for word depth ranging from 0 to 20 s over the movie's course.

**FIGURE 3:**
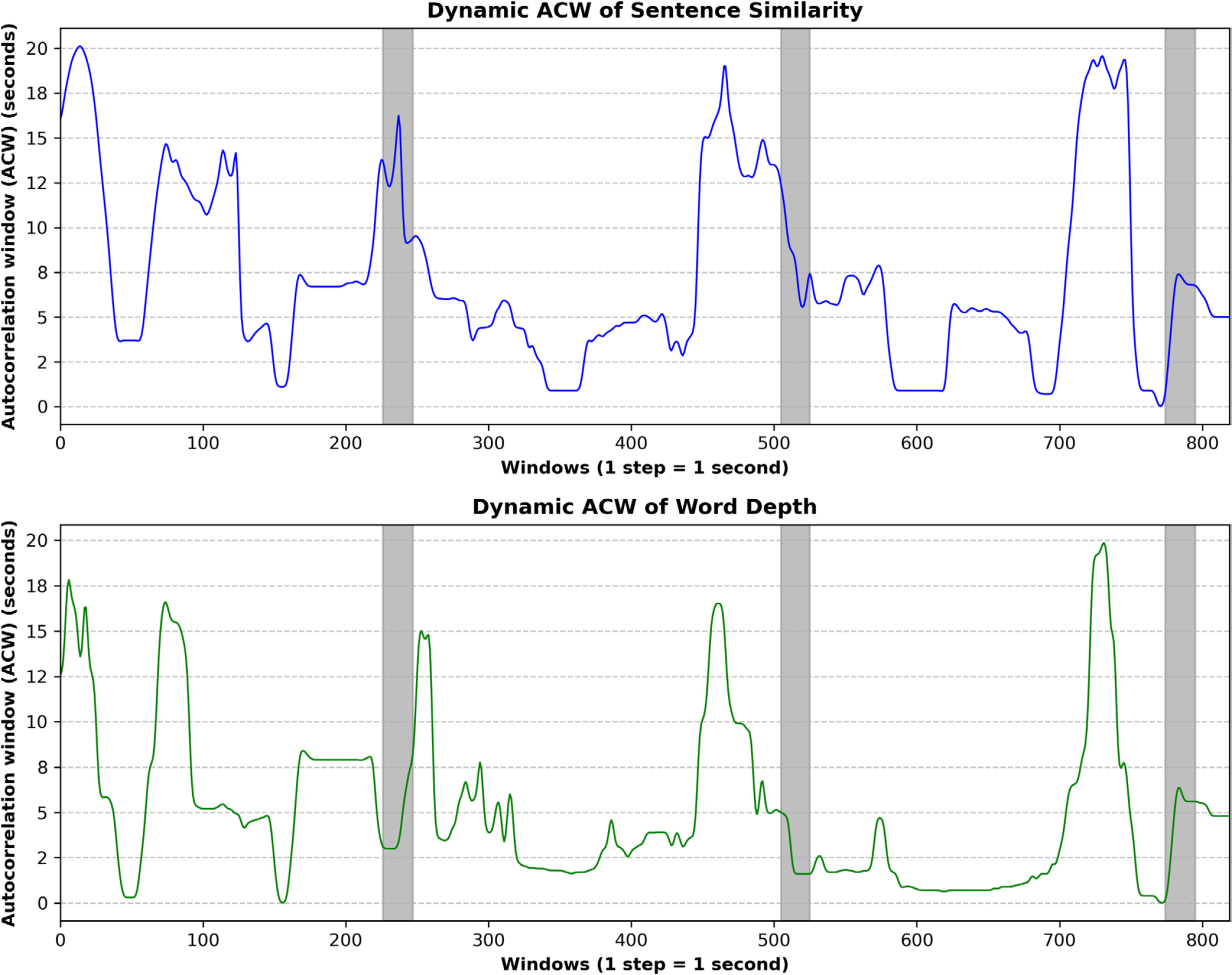
Dynamic ACW of the sentence similarity and word depth time‐series. Using a sliding window of 60 s with a step size of 1 s, we calculated the Dynamic Autocorrelation Windows (ACW) of sentence similarity (blue) and word depth (green) timeseries. The gray vertical bars represent the 20‐s rest periods between the movie's single clips.

In sum, our dynamic ACW results show continuous fluctuations in the timescales (ACW) of both sentence similarity and word depth. Notably, we were able to detect ongoing fluctuations in the semantics' timescales of both words and sentences because we applied a dynamic rather than static analysis of the ACW. In contrast, these ongoing fluctuations in the timescales of the semantic inputs are averaged out ongoing changes in timescales are averaged out by default in a complete run‐based analysis. Figure 3 displays the dynamic ACW results for sentence similarity and word depth.

### 3.3 Brain Timescales—Dynamic ACW of Brain Activity

**FIGURE 4:**
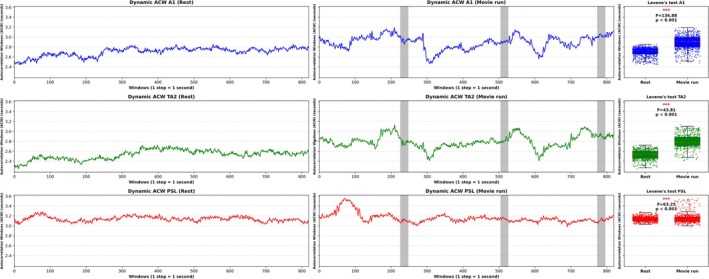
Timescales of brain activity. Dynamic ACW analysis of brain activity in auditory regions A1, TA2, and PSL in the resting‐state and during speech inputs in the movie run. The gray vertical bars represent the 20‐s rest periods between the movie's single clips. (Statistics = Levene's test; significance asterisks *p* < 0.05*, *p* < 0.01**, *p* < 0.001***).

Part of our second aim included revealing continuously changing timescales or INT of the brain's activity. We applied the same dynamic ACW approach previously used for measuring ever‐changing semantic timescales in the brain's auditory regions A1, TA2, and PSL in the resting‐state and during auditory inputs in the movie run (Figure 4). We subsequently applied Levene's test and hypothesized a significantly higher variance of the brain's fluctuating timescales in especially TA2 and PSL as a response to the ever‐varying semantic inputs than in the resting‐state.

*Region A1:* We obtained significantly higher variance in region A1 during the movie's speech inputs than in the resting‐state (*F* = 136.88, *p* < 0.001). This may reflect the auditory processing of the language (words, sentences) as A1 is known to not yet be implicated in semantic language processing.

*Region TA2:* We also obtained significantly higher variance in region TA2 during the movie's speech inputs than in the resting‐state (*F* = 43.81, *p* < 0.001).

*Region PSL:* Finally, we again obtained significantly higher variance in region PSL during the movie's speech inputs than in the resting‐state, although the variance increase remained lower than in regions A1 and TA2 (*F* = 63.25, *p* < 0.001). The dynamic ACW's variability in the PSL region decreased compared to regions A1 and TA2, likely since the Peri‐Sylvian Language area forms part of cortical association areas that are functionally and anatomically relatively far away from the auditory inputs (Rolls et al. 2023, 2022; Glasser et al. 2016).

Together, the dynamic ACW's significantly higher variance during the movie run than in the resting‐state may be related to the varying timescales of the semantic inputs.

### 3.4 Information Transfer From the Ever‐Changing Semantic Inputs (Words, Sentences) to the Ongoing Changes in the Brain Timescales—Transfer Entropy (TE)

Thus far, we have investigated the ever‐changing semantic timescales of sentence similarity and word depth, as well as the varying timescales or intrinsic neuronal timescales or INT of brain activity. Our third and final aim was to examine whether there is information transfer from the ever‐changing timescales of the semantic inputs to the brain's continuously varying timescales. To this end, we measured Transfer Entropy (TE) in the input‐to‐brain direction, specifically from the dynamic ACW of sentence similarity to the brain's dynamic ACW, and from the dynamic ACW of word depth to the brain's dynamic ACW in auditory regions A1, TA2, and PSL. Furthermore, we hypothesized that significant information transfer (TE) would only emerge at the level of continuously varying timescales, as assessed through our dynamic ACW analysis of both the semantic inputs and the BOLD signals. In contrast, we anticipated that input‐to‐brain information transfer would be absent when comparing the raw time‐series of the two semantic inputs (e.g., without calculating their ACW) to the brain's BOLD raw time‐series (e.g., without calculating their ACW).

Methodologically, we used Markov Block Bootstrapping to assess the significance of the Transfer Entropy (TE) values. First, the BOLD time‐series for each subject was divided into blocks, with each block's length matching the subject's average Autocorrelation Window (ACW) calculated during the movie session. These blocks were then shuffled randomly to disrupt the overall temporal order while maintaining the internal dynamics within each block. TE values were computed for 1000 surrogate datasets generated through this process, creating a distribution of TE values under the null hypothesis of no meaningful information transfer. The *p* value was calculated as the number of surrogate TE values that exceeded the original TE value and dividing it by 1000.

*TE from the sentence similarity's timescales to the brain's timescales:* We obtained significant TE (*p* < 0.001) for sentence similarity in all time lags in the A1, TA2, and PSL regions, as shown by the blue bars in Figure 6a.

*TE from the word depth's timescales to the brain's timescales:* We also obtained significant TE for word depth across all time lags, with *p* < 0.001 in the A1 and TA2 regions and *p* < 0.01 in the PSL region, as shown by the green bars in Figure 6a. We can see that the degree of TE for the word depth decreases with increasing lags (green bars) while that is not the case for the cosine similarity/sentences which remain more or less at the same TE level across the different lags. This suggests that words and sentences operate on different timescales which further supports our overall results.

We also computed Transfer Entropy in the input‐to‐brain direction between the semantic inputs' (sentence similarity and word depth) time‐series and the brain's BOLD time‐series, that is, we did not calculate the ACW on their raw time series in either case. Supposing that timescales are key for their information transfer, we hypothesized a lack of input‐to‐brain information transfer on the raw time‐series level.

*TE from sentence similarity time‐series to the brain's BOLD time‐series:* We obtained extremely low and non‐significant TE values close to zero for sentence similarity in all time lags in the A1, TA2, and PSL regions, as shown by the blue bars in Figure 6b.

*TE from word depth time‐series to the brain's BOLD time‐series:* We obtained low and non‐significant TE values for word depth in all time lags in the A1, TA2, and PSL regions, as shown by the green bars in Figure 6b.

Together, the significant TE results in the input‐to‐brain direction on the level of timescales, assessed through our dynamic ACW approach, highlight that the ever‐changing INT of brain activity does not fluctuate randomly but allows an information transfer from the analogously fluctuating timescales of the movie's semantic speech inputs. In contrast, the reverse direction, that is, from brain‐to‐input direction does not show any information transfer (as expected), which speaks to the validity of our significant TE findings. Furthermore, our data showing non‐significant transfer entropy between the raw timeseries of both semantics and brain suggest a key role of timescales in their information transfer.

### 3.5 Whole Brain Analysis‐ Dynamic Inter‐Subject Correlation and Transfer Entropy

Dynamic ISC is generally higher in the raw BOLD time‐series than in the dynamic ACW timeseries: temporal smoothing of the timeseries, as when calculating ACW, is expected to reduce the variations, and as a result, lowers the correlation values across different ROIs. Does this make the brain's dynamic ACW completely unreliable for representing the meaningful information from the semantic inputs? To address this issue, we calculated and compared the ISC of both the raw BOLD time‐series and the ACW time‐series in our data using a correlation matrix of all the 360 regions of interest.

*Inter‐subject Correlations of A1, TA2 and* PSL: We find relatively higher yet comparable ISC across the sliding windows in the BOLD signals and dynamic ACW of A1 (0.231003281 and 0.177462942) and TA2 (0.20969223 and 0.156703124). In the PSL region, ISC of BOLD signals was found to be less than half of that of the dynamic ACW (0.07150348 and 0.196012502). For the lower sensory and associative regions A1 and TA2, ISC seems to be conserved within the ROIs even with the temporal smoothing introduced by the calculation of the dynamic ACW. In higher‐order language associated regions, dynamic ACW possibly captured more stimuli‐related changes leading to higher ISC.

*Triangular Plots*: We also find higher values of ISC between different ROIs for the raw BOLD signals than in the dynamic ACW, though the general pattern of the correlation matrices is similar between the two approaches. Regions showing high ISC in the BOLD signals also show relatively high values in the ISC correlation matrix of dynamic ACW.

*Transfer Entropy*: Transfer Entropy maps of the 360 regions of interests are generally consistent between the Sentence Similarity and Word Depth (Figure 7). The highest TE values are observed in Early Auditory and the Auditory Associative Cortex. Visual Areas such as the MT+ Complex & Neighbors, Ventral Stream Visual Cortex, Dorsal Stream Visual Cortex and Early Visual Cortex also show higher TE values in that order, with more coverage in Sentence similarity than in Word Depth. Parts of higher order regions involved in semantic and speech processing such as the Medial Temporal Cortex, Lateral Temporal Cortex (which contains the Wernicke's area), Insular & Frontal Opercular Cortex and Temporo‐Parieto‐Occipital Junction also have higher TE values, with higher coverage of TOPJ and MTC for Word Depth and IFOC for Sentence Similarity. Small parts of other higher order regions such as the SPC, IPC, PLMCC, PCC, OPFC show high TE, with more coverage of SPC, OPFC in Sentence similarity.

## 4 Discussion

Previous EEG and fMRI studies have provided strong evidence that the brain's INTs, typically assessed during the resting‐state (Wolff et al. 2022; Golesorkhi et al. 2021; Hasson et al. 2015; Yeshurun et al. 2021; Sabanal and Nakagawa 1996; Smith et al. 2022; Luque et al. 2015; Huang et al. 2018; Watanabe et al. 2019; Golesorkhi et al. 2021; Wolff et al. 2022; Klar et al. 2023), and TRWs, which are assessed during stimulus or task states (Hasson et al. 2008; Honey et al. 2012; Murray et al. 2014; Hasson et al. 2015; Honey et al., 2016; Himberger et al. 2018; Yeshurun et al. 2021; Wolff et al. 2022), both represent forms of memory. These forms of memory store either intrinsic information inherent to the brain's own dynamics reflecting TRWs, commonly measured via INTs, or extrinsic information related to external stimuli or task‐driven dynamics. Importantly, recent evidence suggests that INTs and TRWs are interrelated instead of representing two distinct phenomena, with INTs modulating TRWs during stimulus processing or task engagement, so that TRWs can reflect the response or alignment of the brain's ongoing timescales to audiovisual stimulus dynamics (Manea et al., 2022; Watanabe et al. 2019; Ibáñez and Northoff 2024).

Our combined fMRI and input study provides deeper insight into the existing research on INTs and TRWs via three key findings related to the brain's processing of ongoing, naturalistic auditory inputs. In the first part of our study, we demonstrated that human speech inputs, specifically the dialogue between actors in various Hollywood movie clips, are far from static, exhibiting substantial fluctuations in semantic content over time, as captured by measures of word depth and sentence similarity. While it is expected that speech semantics naturally exhibit continuous high fluctuations, explicitly demonstrating these fluctuations serves as an initial indication in support of a more dynamic approach to analyzing both input and brain activity, one that avoids averaging across entire runs, as is common in traditional studies of INTs (Wolff et al. 2022; Golesorkhi et al., 2021a; Hasson et al. 2015; Yeshurun et al. 2021; Sabanal and Nakagawa 1996; Smith et al. 2022; Luque, 2015; Huang et al. 2018; Watanabe et al. 2019; Golesorkhi et al., 2021; Klar et al. 2023) and TRWs (Hasson et al. 2008; Honey et al. 2012; Murray et al. 2014; Hasson et al. 2015; Honey et al., 2016; Himberger et al. 2018; Yeshurun et al. 2021; Wolff et al. 2022).

Second, these fluctuations were not limited to the two raw semantic time‐series; they also emerged at the level of the dominant, or primary, timescales of word depth and sentence similarity, as measured through our dynamic ACW analyses. By “driving” or “primary” timescale, we refer to the fact that while both semantic signals and brain activity (as assessed via ACW) contain a wide range of frequencies, and therefore multiple timescales, the ACW measure is primarily influenced by the dominant frequencies, which contribute most to the signal's variance and autocorrelation (Bassingthwaighte et al., 1994; Williams 1997; Klar et al. 2023; Catal et al., 2024). As a result, ACW predominantly reflects timescales that are particularly rich in memory or information.

**FIGURE 5:**
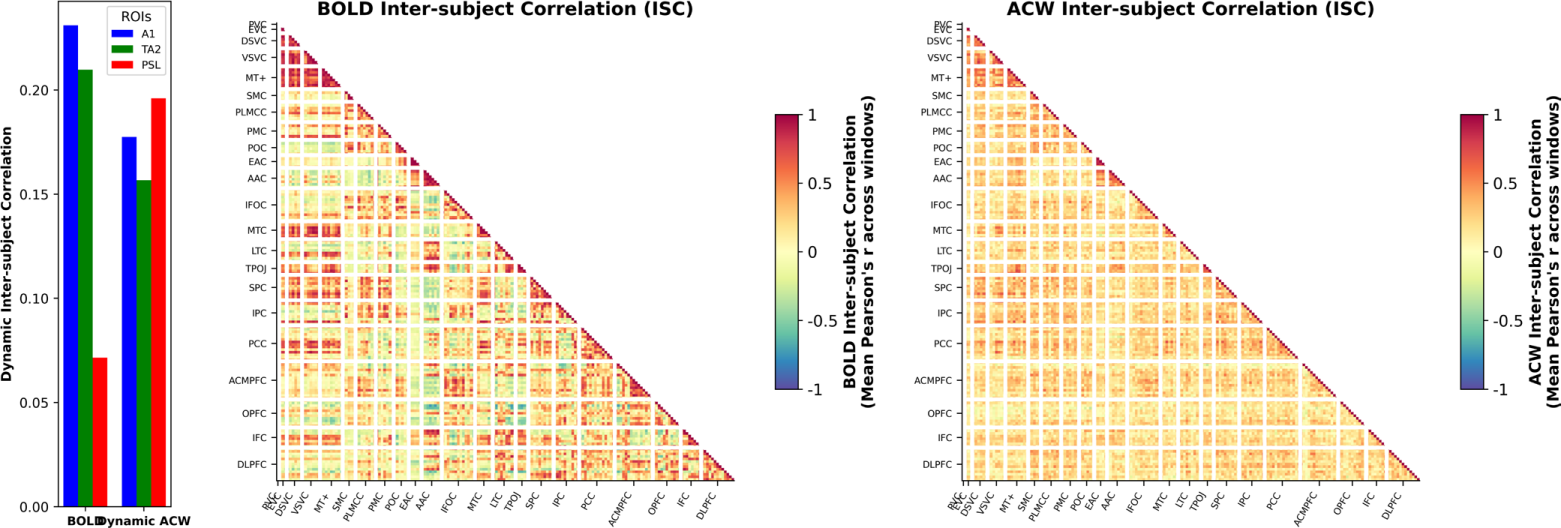
Inter‐subject correlation. We measured the ISC for every 60 s overlapping windows for the BOLD timeseries and the dynamic ACW timeseries. (a) We observed that ISC is prevalent in both BOLD timeseries and the dynamic ACW for the ROIs A1, TA2 and PSL, although it is lower for dynamic ACW. (b) We made correlation matrix across all ROIs using the ISC values from the sliding windows, on the BOLD timeseries (left) and the dynamic ACW timeseries (right). Lower correlation values are present across the plot in dynamic ACW than in raw BOLD fluctuations. ROIs were grouped into the functional networks according to “supporting information Neuroanatomical Results” of Glasser et al. (2016). (AAC: auditory association cortex; ACMPFC: anterior cingulate & medial PFC; DLPFC: dorsolateral prefrontal cortex; DSVC: dorsal stream visual cortex; EAC: early auditory cortex; EVC: early visual cortex; IFC: inferior frontal cortex; IFOC: insular & frontal opercular cortex; IPC: inferior parietal cortex; LTC: lateral temporal cortex; MT+: MT+ complex & neighbors; MTC: medial temporal cortex; OPFC: orbital & polar frontal cortex; PCC: posterior cingulate cortex; PLMCC: paracentral lobular & mid cingulate; PMC: premotor cortex; POC: posterior opercular cortex; PVC: primary visual cortex; SMC: somatosensory & motor cortex; SPC: superior parietal cortex; TPOJ: temporo‐parieto‐occipital junction; VSVC: ventral stream visual cortex).

We compare the persistence of meaningful information of the naturalistic stimuli that is captured by the raw BOLD fluctuations and with that of the dynamic ACW timeseries using Inter‐subject Correlation (ISC). ISC has been shown to be able to differentiate between attentional states such as movie watching or speech (Hasson et al. 2008, 2010; Rosenkranz et al. 2021), making it a reliable tool to test whether features of naturalistic input are also reflected in the dynamic ACW of participants. We find lower yet comparable average ISC between dynamic ACW and the BOLD fluctuations across the three main regions of interest of our study (Figure 5a). A similar pattern is observed when examining the correlation matrices of ISC between different ROIs (Figure 5b), suggesting that the dynamic ACW preserves region‐specific temporal patterns of neural activity. These findings indicate that, although dynamic ACW represents a derived temporal feature rather than raw signal amplitude, it still captures shared neural dynamics across participants. This supports the interpretability of ACW as a meaningful measure of intrinsic timescales in naturalistic conditions. Notably, the slightly lower ISC in ACW compared to raw BOLD may reflect the additional processing involved in estimating autocorrelation structures, which can introduce participant‐specific variability while still retaining the core features of stimulus‐driven temporal structure.

**FIGURE 6:**
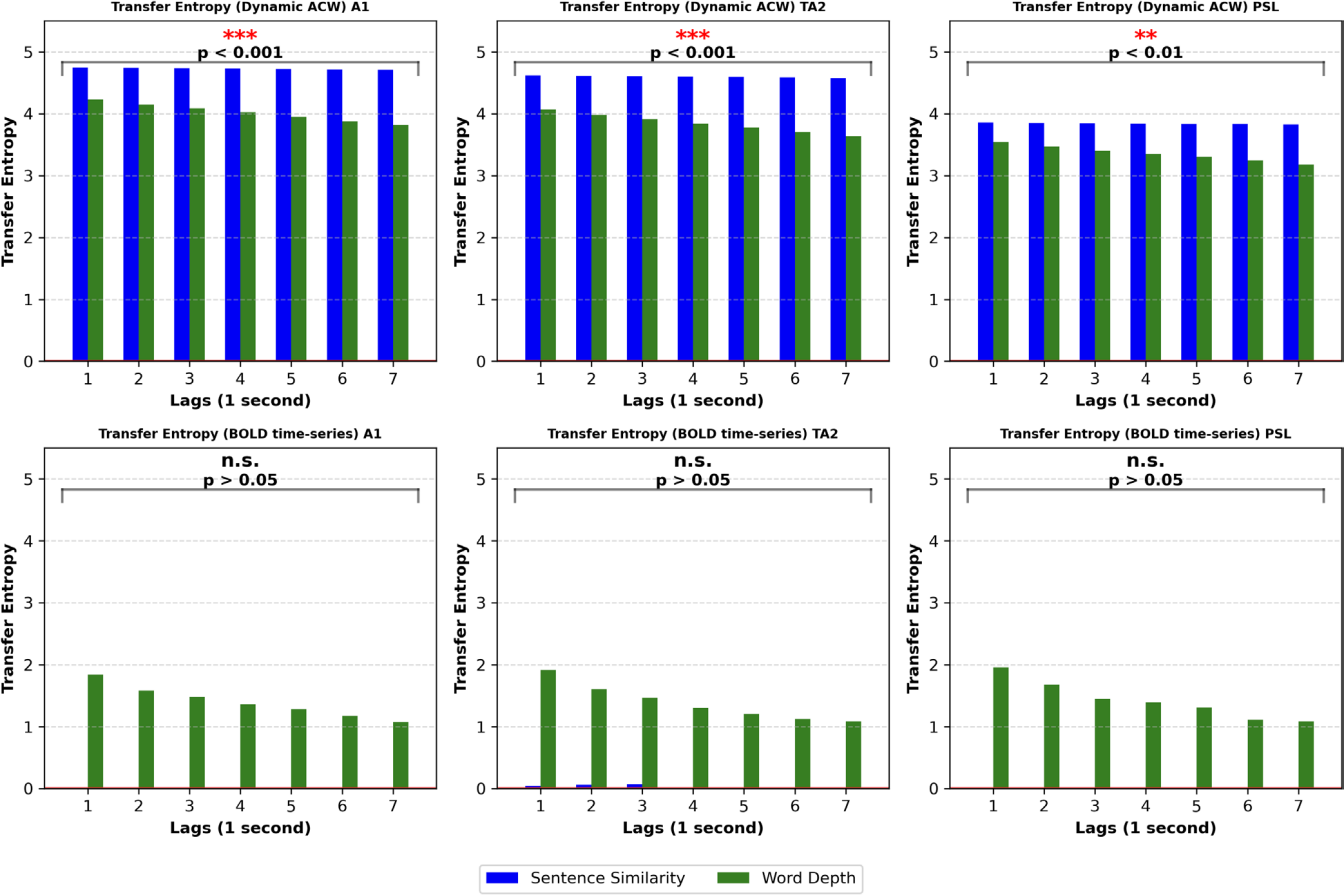
Transfer entropy. Information transfer (Transfer Entropy) from semantic input to brain timescales during movie‐watching. (a) We computed Transfer Entropy in the input‐to‐brain direction between the semantic inputs' (sentence similarity and word depth) and the brain's dynamic ACW. (b) We also computed transfer entropy in the input‐to‐brain direction between the semantic inputs' (sentence similarity and word depth) time‐series and the brain's BOLD time‐series. (Statistics = Markov bootstrap procedure to estimate the statistical significance of Transfer Entropy; significance asterisks *p* < 0.05*, *p* < 0.01**, *p* < 0.001***).

**FIGURE 7:**
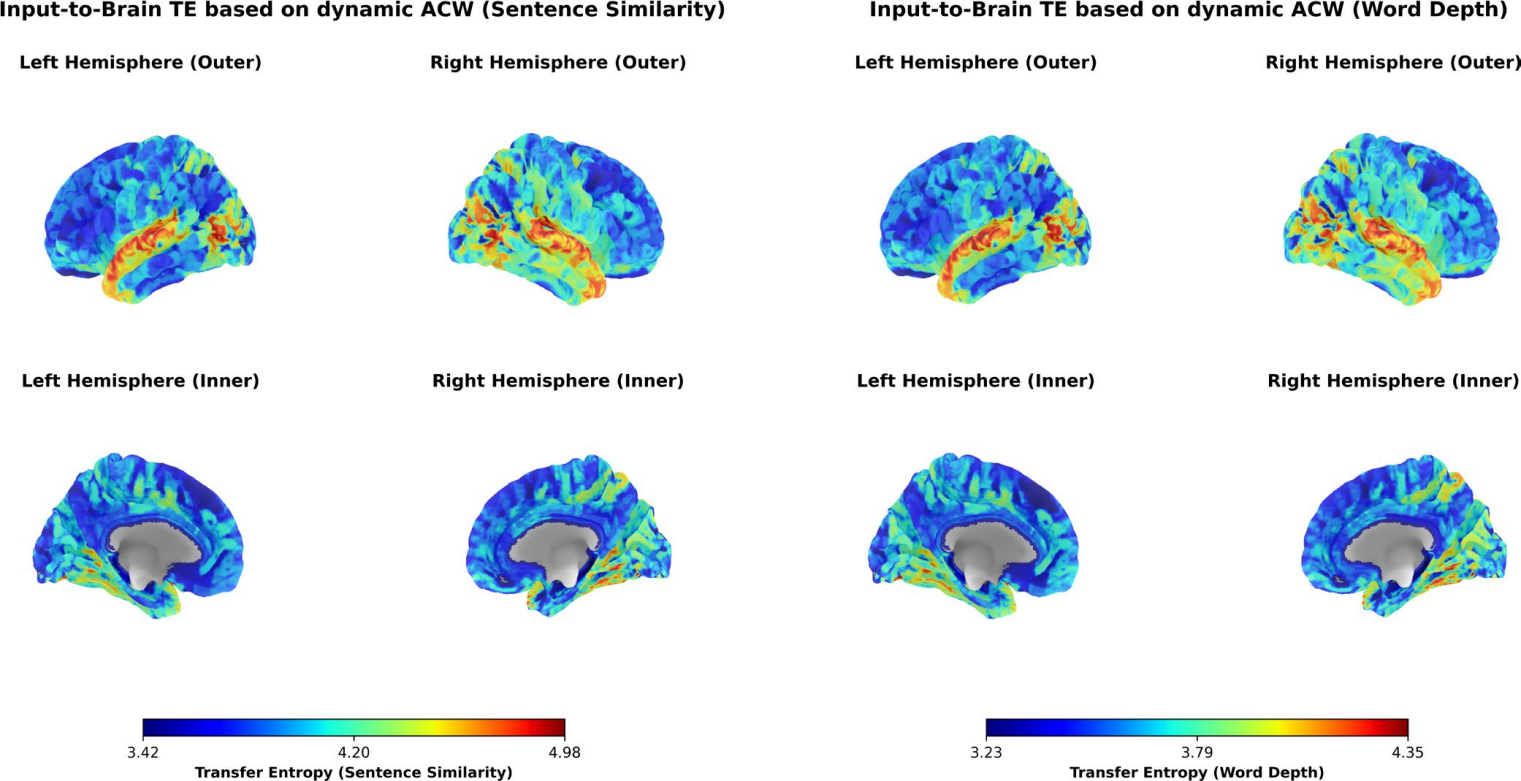
Brain surface map of transfer entropy. We computed Transfer Entropy for the first lag from the dynamic ACW of Sentence Similarity (above) and Word Depth (below) to all 360 regions of interest of the Glasser atlas. The highest TE values are observed in the Early Auditory and Auditory Association Cortex, with visual areas such as the MT+ Complex, Ventral Stream Visual Cortex, Dorsal Stream Visual Cortex, and Early Visual Cortex also showing elevated TE. Higher‐order regions in the temporal lobe such as the Lateral and Medial Temporal Cortex involved in semantic and speech processing exhibit high TE values, with differences in coverage between the two inputs, particularly in TPOJ. Early visual regions show TE levels comparable to auditory regions, reflecting the integration of visual cues with auditory processing during language comprehension.

**FIGURE 8:**
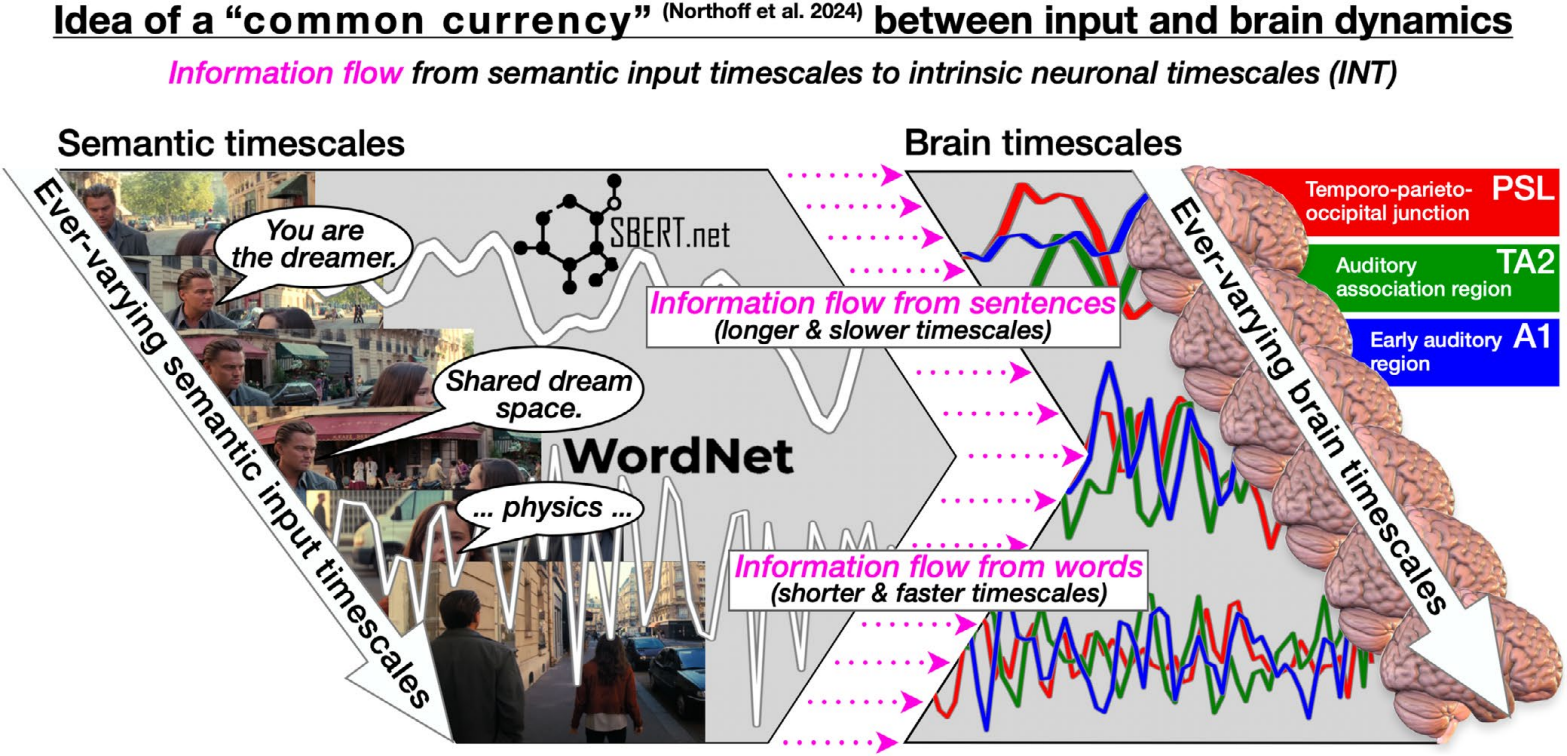
Common currency framework. Illustration of the “common currency” framework for linking semantic input timescales and brain dynamics. Semantic timescales (left), ranging from faster timescales associated with words to slower timescales associated with sentences, align with brain timescales (right) at corresponding speeds. Information transfer occurs from faster semantic timescales to faster brain timescales and from slower semantic timescales to slower brain timescales, showing the dynamic alignment between linguistic inputs and neuronal processes.

The timescales richest in information are mostly the slower ones, both in many natural phenomena, such as auditory inputs (Voss and Clarke 1975; Sabanal and Nakagawa 1996; Luque, 2015), which often exhibit pink, brown, or black noise where power is concentrated in the slower frequency ranges rather than being uniformly distributed across the spectrum as in white noise, and in brain activity (Linkenkaer‐Hansen et al. 2001; Smith et al. 2022; Huang et al. 2018; Wolff et al. 2022; Klar et al. 2023; Northoff et al. 2023). This is also demonstrated by the power spectra for sentence similarity, word depth, and BOLD time series in Figures S1 and S2, which highlight the predominance of signal power or variance in slower, as opposed to faster, frequencies. The temporal structure observed in the dynamic ACW of both word depth and sentence similarity, as well as in brain activity, suggests that ACW is not merely capturing random noise. Rather, it reflects slow and powerful fluctuations rich in memory or informational content that vary over time in accordance with the ever‐changing semantic richness of speech, such as from concrete to abstract words, in the movie clips.

Third and most importantly, our analysis deliberately focused on semantic content rather than on the full auditory input, which has been implicitly present in previous studies on TRWs (Hasson et al. 2008; Honey et al. 2012; Murray et al. 2014; Hasson et al. 2015; Honey et al. 2016; Himberger et al. 2018; Yeshurun et al. 2021; Wolff et al. 2022). In previous TRW studies, the full auditory input, including non‐speech elements such as music or environmental landscape and urban sounds, remains indistinguishable from speech semantics, precisely because these studies analyzed only brain dynamics. Since brain responses naturally reflect the entire auditory input, it is impossible to determine whether, and to what extent, the brain specifically tracks the temporal structure of semantic information. Without explicitly modeling semantic input, any observed TRWs could reflect general auditory dynamics rather than the more specific processing of meaningful language content and its semantics by themselves. While this limitation also applies to our study, namely, that we analyzed brain timescales without isolating the BOLD signal components specifically driven by speech semantics, we partially addressed it by explicitly analyzing semantic input timescales and their information transfer to brain timescales. By focusing exclusively on the semantic properties of speech, namely, word depth and sentence similarity, we were able to isolate and examine the auditory input's semantic contribution, which in turn was part of the significant input‐to‐brain information transfer measured through TE. Importantly, information transfer from semantics to the brain occurred only at the level of the driving timescales, as assessed through our dynamic ACW analyses of the semantic inputs and the BOLD signals. This transfer was absent when considering the raw input and brain time‐series variability, further highlighting the relevance of slow and powerful timescales that are particularly rich in information.

The observed Transfer Entropy (TE) map of semantic information flow shows high TE not only in the expected auditory and associative cortices but also engages visual, medial temporal, and fronto‐parietal regions (Figure 7), indicative of a multimodal semantic network (Doerig et al. 2022; van Wassenhove et al. 2005; Lambon Ralph 2013; Fernandino et al. 2022, Fernandino et al. 2022; Tong et al. 2022). A comparison with macaque and human semantic networks using meta‐analyses of fMRI studies can be seen in figs. 7 and 9 of Binder et al. (2009). Elevated TE in medial and lateral portions of the temporal cortex supports their role in accessing conceptual knowledge and integrating semantic information (Zahn et al. 2007). Interestingly, we also observed high TE in early visual regions, at levels comparable to the upper temporal lobe, on our cortical surface plots. This likely reflects the complementary role of visual perception in speech processing: listeners' perception of spoken language is influenced by visual cues from the moving lips of speakers, an unconscious or conscious form of lip reading (Massaro and Stork 1998; van Wassenhove et al. 2005). This is consistent with the significant functional connectivity between early visual and auditory association regions reported in the HCP MMP atlas (Rolls et al. 2022, 2023).

What do our findings imply on a theoretical level? Here, we compared two naturally very different phenomena: the semantics of human speech and the corresponding response of brain as measured from BOLD signals. We demonstrated that it is possible to bridge this gap by revealing that these apparently distinct phenomena enable information transfer through their underlying and continuously varying timescales. Bridging this gap by uncovering shared dynamics in the timescales of different phenomena, such as audiovisual inputs and brain activity, is a form of “common currency” proposed by the Temporo‐Spatial Theory of Consciousness (TTC). This common currency allows for connecting behavioral‐psychological as well as audiovisual stimuli processes with neuronal dynamics (Northoff et al. 2023; Goheen et al. 2024; Chis‐Ciure et al. 2024; Northoff et al. 2020; Kolvoort et al. 2020; Northoff et al. 2025). Our combined semantic input and fMRI study adheres to this common currency approach by implicating a form of brain‐to‐input alignment that enables significant degrees of information transfer from the inputs' dynamics to the brain's dynamics through their shared timescales (Figure 8).

We conducted a series of control analyses to support the theoretical inferences drawn above. First, we found no significant TE in the reverse brain‐to‐input direction across the three auditory regions investigated, as shown in Figure S3. Second, we individually shuffled the time‐series of the two semantic inputs before applying timescale assessment using our dynamic ACW approach. The timescales derived from the shuffled sentence similarity and word depth time series did not produce any information transfer to the brain's timescales or INT, as shown in Figure S4. While we conducted control analyses to support our conclusions, there are limitations in our study that should be noted and are discussed in the limitations section.

Finally, we shall briefly return to the broader theoretical framework in which our study and results are set. Our investigation of shared timescales between auditory (semantic) inputs and the BOLD signals follows the methodological common currency strategy of the Temporo‐spatial Theory of Consciousness (TTC) (Northoff et al. 2023; Goheen et al. 2024; Chis‐Ciure et al. 2024; Northoff et al. 2025). The TTC posits, and empirical studies have supported, that (1) brain dynamics do not necessarily differ from the dynamics observed in other natural phenomena (Cocchi et al. 2017; Northoff 2024); (2) the brain's input processing utilizes its intrinsic timescales to align with the input's timescales (Northoff et al. 2023; Klar et al. 2023), which amounts to (3) shared timescales between input and brain as their “common currency” (Northoff et al. 2020, 2025). Future studies should aim not only to integrate analysis of both input dynamics and brain dynamics, but also to include the output dynamics, for example, psychological and behavioral data; this serves to reveal how the brain's alignment of its neuronal timescales to the inputs' timescales translates into outputs like conscious experience and behavior (see Chuipka et al. 2025 for a first step in this direction).

## 5 Limitations

We now discuss several limitations of our study. First, we acknowledge that the 60‐s window used for our dynamic ACW analysis is relatively long for capturing the semantic content of the movie's speech, particularly when using SBERT and WordNet methods, as well as for analysing the brain's BOLD signal. Within a single minute of the movie, multiple sentences and a substantially greater number of words are presented. A shorter window, such as 5 to 8 s, sufficient to capture approximately two pairs of sentences for semantic similarity analysis, would likely be more appropriate. However, three key factors constrained us to a minimum window length of approximately 60 s. First, the inherently sluggish nature of the BOLD signal (Logothetis 2008), combined with the particularly low fMRI sampling rate of 1 Hz in the 7T HCP dataset, necessitates the use of longer windows. Shorter windows would require modalities with higher temporal resolution, such as EEG or MEG. Second, a sufficient number of sampling points is required to reliably compute measurements like the ACW with an adequate signal‐to‐noise ratio, which is unachievable with very short windows containing only a few data points in fMRI. Third, short windows also limit the lowest frequency components that can be captured. For instance, even a 30‐s window would restrict the lower bound of the frequency spectrum to around 0.1 Hz, assuming at least three cycles per window are needed for accurate ACW estimation (Huang et al. 2018). Consequently, important information in the sub 0.1 Hz frequency range (Logothetis 2008) would be lost.

The sluggishness of the BOLD signal (Logothetis 2008) may also be responsible for the non‐significant TE at the level of raw time‐series. Semantic content, such as individual words or sentences, evolves on timescales much shorter than the approximately 10‐s response window dictated by the hemodynamic response function (Li et al. 2019), while the alignment between the longer timescales of semantic fluctuations and the BOLD signal's resolution could have been responsible for the detection of significant information transfer at the level of continuously varying timescales.

Another related limitation concerns the treatment of each 2–4‐min clip from the approximately 15‐min movie as a quasi‐independent unit of analysis for computing input‐to‐brain Transfer Entropy. By assuming that each clip represents an independent segment of narrative, we expect that information transfer from the timescales of speech semantics will be detectable in the timescales of brain activity during the corresponding period. However, we find that the TE values show higher *p* values and start to become insignificant for some ROIs, such as PSL in the third movie, or TA2 in the second movie for Word Depth (Figure S5). The fourth and the last movie, which is only 82 s long, shows lower and completely insignificant TE values for almost all the cases. This is because when we use a 60 s window, only 12 samples survive with the sliding windows. Transfer Entropy, when estimated using k‐nearest neighbours (kNN) methods such as the KSG algorithm employed in our study, relies on the assumption that local probability patterns detected at the level of nearest neighbours are representative of the global structure of the data (Lord et al. 2018). This assumption breaks down when dealing with small datasets derived from systems with complex underlying geometries, such as the human brain or bidirectional encoders (Lord et al. 2018). As a result, the kNN‐based Transfer Entropy estimation is highly sensitive to the size of the dataset, showing significant variability when sample sizes range from 100 to 1000, and only stabilizing when applied to datasets with approximately 1 million samples (Lord et al. 2018).

A further limitation concerns the choice of sliding window length for estimating dynamic ACW and subsequent Transfer Entropy. While shorter windows provide sufficient temporal resolution to capture sentence‐ or word‐level dynamics, very short windows can lead to noisy TE estimates due to limited data points. Conversely, very long windows average over multiple sentences or words, blurring the stimulus‐related temporal patterns and reducing TE values, often to the point of losing statistical significance. To empirically illustrate this effect, we conducted supplementary analyses using window durations up to 540 s, which revealed a progressive decline in TE values and eventual loss of significant interactions across ROIs (Figure S6).

Another potential limitation is the fact that subjects were passively watching movie clips rather than engaging in a real‐time dialogue with another person. A future study might address this by using electroencephalography (EEG) in a more natural setting, such as allowing non‐scripted dialogues between subjects (Gonzalez et al. 2024). We hypothesize that the information transfer, as measured by Transfer Entropy from one subject's speech and semantics to the brain dynamics of a listening subject, would be higher in the context of a real‐time dialogue, where subjects constantly alternate roles between speaking and listening. During movie‐watching in our assessed HCP dataset, subjects were only passively listening and did not engage in any active role, such as responding or providing answers. As a result, constant high levels of attention were neither required nor guaranteed, which may have reduced the information transfer from the semantic inputs' timescales to the brain's timescales.

We limited our main analysis to three regions focusing on a quasi‐proof‐of‐principle approach, namely to demonstrate the connection between semantic and neuronal timescales. This, of course, leaves many details unresolved, such as the potential involvement of other regions like the anterior temporal lobe and the posterior superior temporal sulcus, which are well known to play a role in semantic processing (Pulvermüller 1999, 2018). Future studies will need to address this. Additionally, we were unable to account for various features of speech, such as variation in loudness, emphasis, intonation, and so on, which often indicate topic changes, surprising speech acts, or unexpected verbal information. These features may independently activate all three regions we selected (see, for example, Uppenkamp et al. 2006). Future research is needed to explore how these factors influence the degree of information transfer from speech to the brain.

Another unexamined dimension is the syntactic structure of the speech. Syntax, like semantics, may fluctuate over time, potentially in ways that differ from semantic fluctuations, and it is conceivable that these temporal dynamics engage distinct cortical areas (e.g., Broca's and Wernicke's regions) or modulate timescale alignment in different ways. Although beyond the scope of our current analysis, incorporating dynamic syntactic measures alongside semantic features could provide a more complete picture of how multiple linguistic subsystems jointly shape brain activity during naturalistic language comprehension.

A further limitation concerns the separation of semantic input dynamics from the broader auditory and visual contextual properties of the movie. Our current analysis does not explicitly account for the contribution of non‐speech auditory features (e.g., music and environmental sounds) or concurrent visual information to the observed brain dynamics. Future studies could address this by combining semantic analyses with independent measurements of auditory and visual input dynamics and potentially subtracting these from the semantic signal to obtain a more “pure” semantic time‐series. While this could help isolate semantic contributions more precisely, it remains technically challenging to fully disentangle speech‐driven activity, particularly within auditory cortices, from that elicited by other co‐occurring sensory features in complex, naturalistic stimuli.

From a semantic perspective, one might ask what type of meaning is addressed in our study. Cosine similarity compares sentences with each other, focusing on a rather coarse‐grained layer of semantic meaning, potentially influenced by semantic priming or, conversely, semantic surprise, both of which can elicit changes in neuronal activity (see, e.g., Grisoni et al. 2016; Grisoni et al. 2021). However, the meaning of sentences or words can be measured at a more fine‐grained level, with related cosine similarity measures of semantic similarity as a second‐order metric within a multidimensional semantic space. It is also possible to map meaning and semantic similarity in the brain at finer scales using, for example, contextual vector representations (Carota et al. 2017), word depth (Carota et al. 2021), or associations between language and real‐world entities (Carota et al. 2023; Fernandino et al., 2022). Such approaches suggest that different cortical areas play key roles in processing various types of semantic similarity. This remains an area for future investigation.

## 6 Conclusion

In summary, our combined analysis of semantic input and fMRI data provides preliminary evidence that the brain aligns its intrinsic neuronal timescales with the timescales of human speech semantics to facilitate input‐to‐brain information transfer and flow. This alignment likely plays a role in enabling the comprehension of auditory semantic content. Based on a range of control and shuffling analyses, we tentatively conclude that intrinsic timescales are a key factor in the transfer of information from speech semantics to neuronal activity. Our findings can be interpreted within the framework of temporo‐spatial alignment (Northoff et al. 2023), which we define as the moment‐to‐moment matching of the brain's intrinsic dynamics with those of external stimuli.

Notably, the concept of temporo‐spatial alignment differs from traditional frameworks such as encoding, which emphasizes the transformation of stimuli into internal neural representations, and entrainment, which refers to the synchronization of neural activity with rhythmic inputs. In contrast, alignment emphasizes shared dynamic trajectories between the BOLD signals and external stimuli, without necessitating periodicity of inputs (as in entrainment) or representational transformation (as in encoding). Within this perspective, semantic timescales and neuronal timescales evolve in parallel, facilitating more efficient information transfer, as supported by our Transfer Entropy results. Our study adds to a growing body of research suggesting that the brain leverages the dynamics of external inputs and its own activity through their mutually shared timescales to support cognitive functions such as the comprehension of semantic information.

## Author Contributions

**Saket Kumar:** conceptualization, formal analysis, investigation, methodology, validation, visualization, writing – review and editing. **Philipp Klar:** conceptualization, investigation, methodology, supervision, validation, visualization, writing – original draft, writing – review and editing. **Yasir Çatal:** conceptualization, methodology, supervision, validation, writing – review and editing. **Han‐Jen Chang:** methodology, software, validation. **Friedemann Pulvermüller:** supervision, validation, writing – review and editing. **Georg Northoff:** conceptualization, funding acquisition, methodology, project administration, supervision, validation, writing – review and editing.

## Ethics Statement

This study utilizes publicly available data from the Human Connectome Project. The data were collected from healthy adult volunteers who provided informed consent for the use of their data for research purposes. No personally identifiable information is included in the dataset. The study involved secondary analysis of publicly available data, for which ethics approval was obtained at the University of Ottawa Institute of Mental Health Research (REB Number: 2021002).

## Conflicts of Interest

The authors declare no conflicts of interest.

## Supporting information


**Figure S1:** Power‐spectral density of semantics.
Power‐Spectral Density of Sentence Similarity time‐series (blue) and Word Depth time‐series (green), calculated using Welch's method.

**Figure S2:** Power‐Spectral Density of BOLD time‐series.
Average Power Spectral Density of BOLD time‐series calculated using Welch's method, across 182 subjects after linear detrending and bandpass filtering (0.05–0.5 Hz), shown for Rest (left) and Movie Watching (right) conditions. Regions of interest: A1 (blue), TA2 (green), and PSL (red).

**Figure S3:** Information does not flow from Brain to Semantics.
Information transfer (Transfer Entropy) from brain timescales to semantic input during movie‐watching.
(a) We computed Transfer Entropy in the brain‐to‐input direction between the brain's and the semantic inputs' (sentence similarity and word depth) dynamic ACW. (b) We also computed Transfer Entropy in the brain‐to‐input direction between the brain's BOLD time‐series and the semantic inputs' (sentence similarity and word depth) time‐series. (Statistics = Markov bootstrap procedure to estimate the statistical significance of Transfer Entropy; significance asterisks *p* < 0.05*, *p* < 0.01**, *p* < 0.001***).

**Figure S4:** Shuffling of Semantics disrupts information flow to the Brain.
Information transfer (Transfer Entropy) from semantic input, shuffled using Markov Block Bootstrapping, to brain timescales during movie‐watching.
(a) We computed Transfer Entropy in the input‐to‐brain direction between the shuffled semantic inputs' (sentence similarity and word depth) and the brain's dynamic ACW. (b) We also computed Transfer Entropy in the input‐to‐brain direction between the shuffled semantic inputs' (sentence similarity and word depth) time‐series and the brain's BOLD time‐series. (Statistics = Markov bootstrap procedure to estimate the statistical significance of Transfer Entropy; significance asterisks *p* < 0.05*, *p* < 0.01**, *p* < 0.001***).

**Figure S5:** Transfer entropy for Individual movies.
Information transfer (Transfer Entropy for the first lag) from semantic input to the brain during movie watching, analyzed for segments belonging to each of the four individual movie clips within the full 15‐min session. Columns show results for each clip. Rows 1–2: TE from Sentence Similarity to BOLD signals at the level of dynamic ACW (row 1) and raw fluctuations (row 2). Rows 3–4: TE from Word Depth to BOLD signals at the level of dynamic ACW (row 3) and raw fluctuations (row 4). Within each subplot, bars represent TE values for each ROI: A1 (blue), TA2 (green), PSL (red). The *y*‐axis shows TE values, and the *x*‐axis shows the regions of interest. (Statistics = Markov bootstrap procedure to estimate the statistical significance of Transfer Entropy; significance asterisks *p* < 0.05*, *p* < 0.01**, *p* < 0.001***).

**Figure S6:** Transfer Entropy for very long windows.
Information transfer (Transfer Entropy for the first lag) from semantic input to the brain at the level of dynamic ACW, calculated using sliding windows of lengths 60–540, with corresponding *p* values. Each color map has 9 rows (corresponding to the 9 window sizes) and 3 columns (ROIs: A1, TA2, PSL).
Top color maps: TE values from Sentence Similarity (green, left) and Word Depth (blue, right) to BOLD signals at the level of dynamic ACW. Color maps below: corresponding *p* values for the TE estimates; *p* values were calculated using Markov‐block bootstrapping, with significance indicated as: *p* < 0.001 (red), *p* < 0.01 (orange), *p* < 0.05 (yellow), *p* > 0.05 (grey, non‐significant).

## Data Availability

The data that support the findings of this study are openly available in “Semantic and Brain Dynamics” (Kumar and Klar 2025) at http://doi.org/10.17632/6zv4bk77gf.2.
