# Supplementary figures and images for: From Speech Semantics to Brain Activity—Timescales Are Key in Their Information Transfer
Source: Hum Brain Mapp. 2025 Oct 25;46(16):e70379. doi: 10.1002/hbm.70379 (PMC12553116; doi:10.1002/hbm.70379)

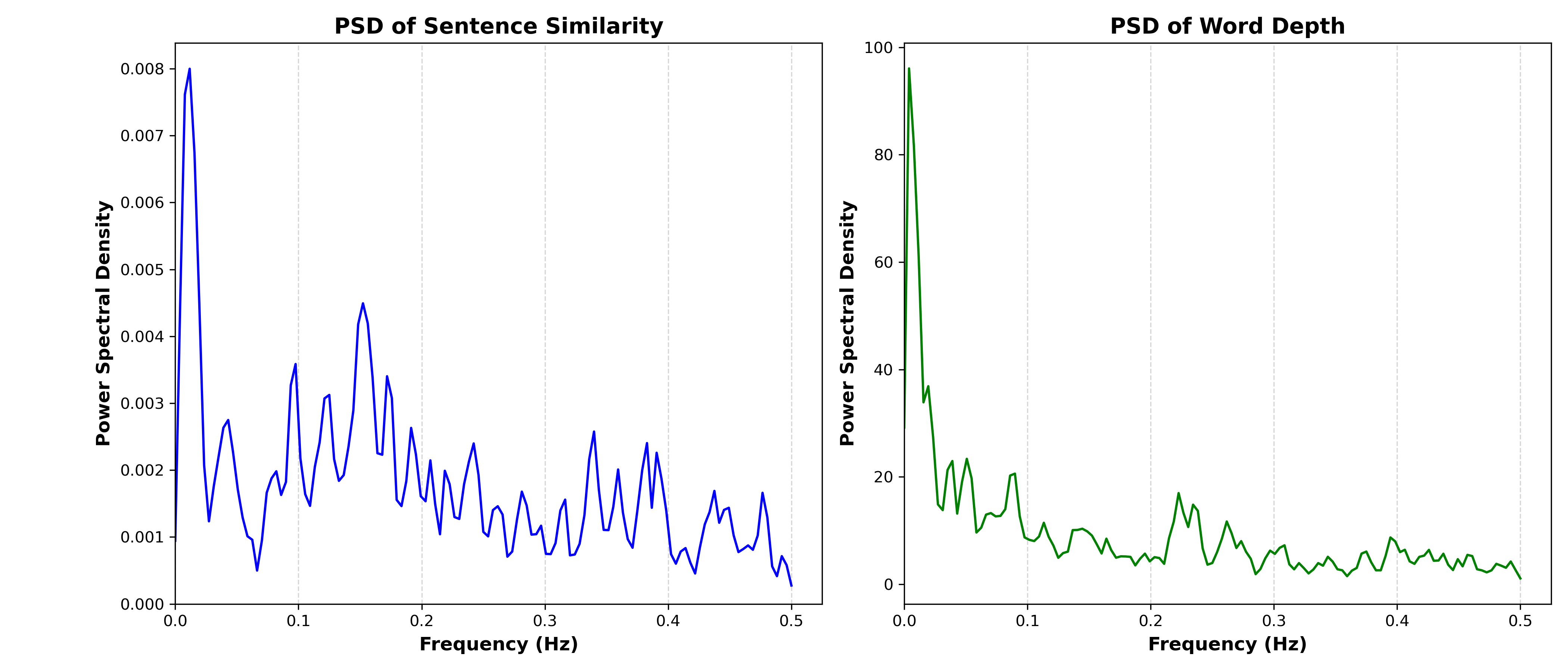

Supplement: Supplementary file 1 — Figure S1: Power‐spectral density of semantics. Power‐Spectral Density of Sentence Similarity time‐series (blue) and Word Depth time‐series (green), calculated using Welch's method. [file HBM-46-e70379-s002.tiff]

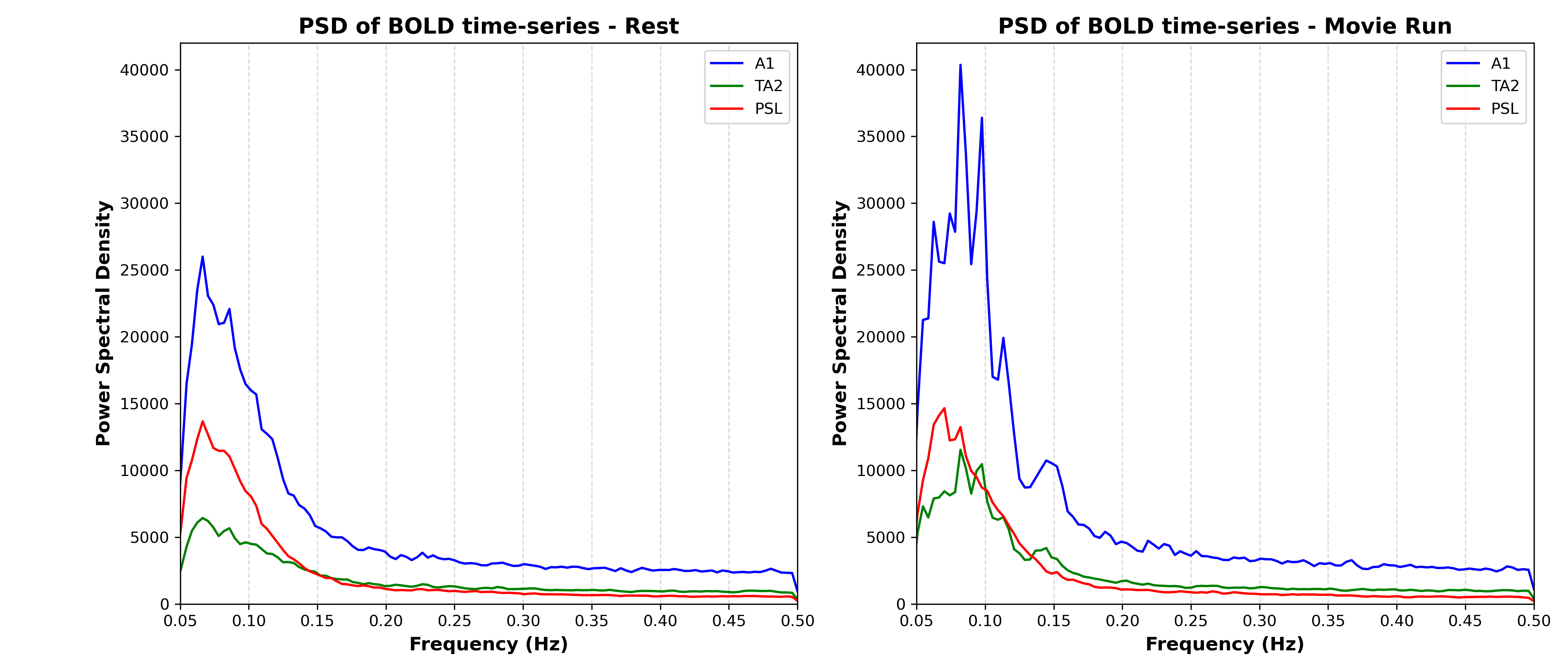

Supplement: Supplementary file 2 — Figure S2: Power‐Spectral Density of BOLD time‐series. Average Power Spectral Density of BOLD time‐series calculated using Welch's method, across 182 subjects after linear detrending and bandpass filtering (0.05–0.5 Hz), shown for Rest (left) and Movie Watching (right) conditions. Regions of interest: A1 (blue), TA2 (green), and PSL (red). [file HBM-46-e70379-s006.tiff]

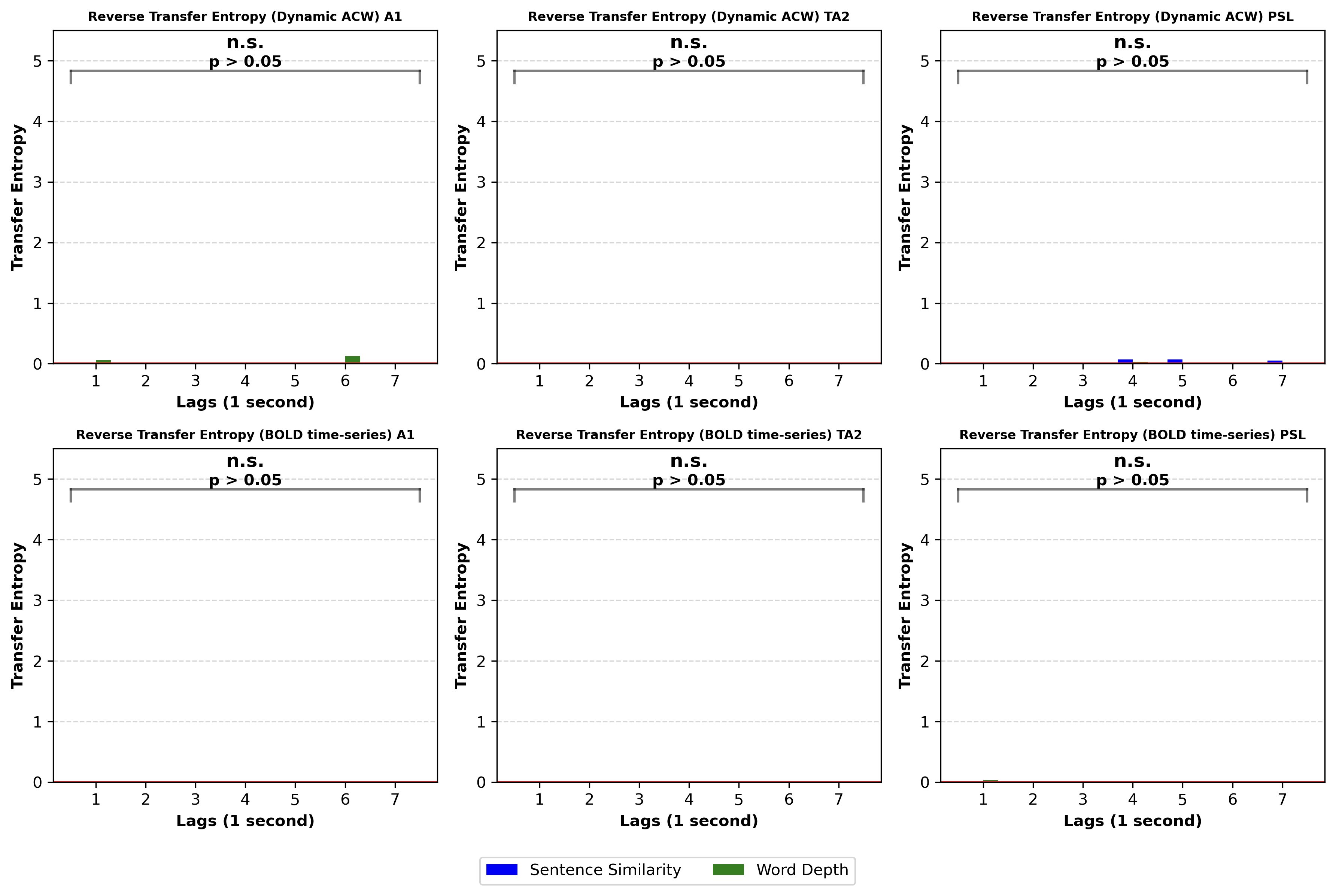

Supplement: Supplementary file 3 — Figure S3: Information does not flow from Brain to Semantics. Information transfer (Transfer Entropy) from brain timescales to semantic input during movie‐watching. (a) We computed Transfer Entropy in the brain‐to‐input direction between the brain's and the semantic inputs' (sentence similarity and word depth) dynamic ACW. (b) We also computed Transfer Entropy in the brain‐to‐input direction between the brain's BOLD time‐series and the semantic inputs' (sentence similarity and word depth) time‐series. (Statistics = Markov bootstrap procedure to estimate the statistical significance of Transfer Entropy; significance asterisks p < 0.05*, p < 0.01**, p < 0.001***). [file HBM-46-e70379-s003.tiff]

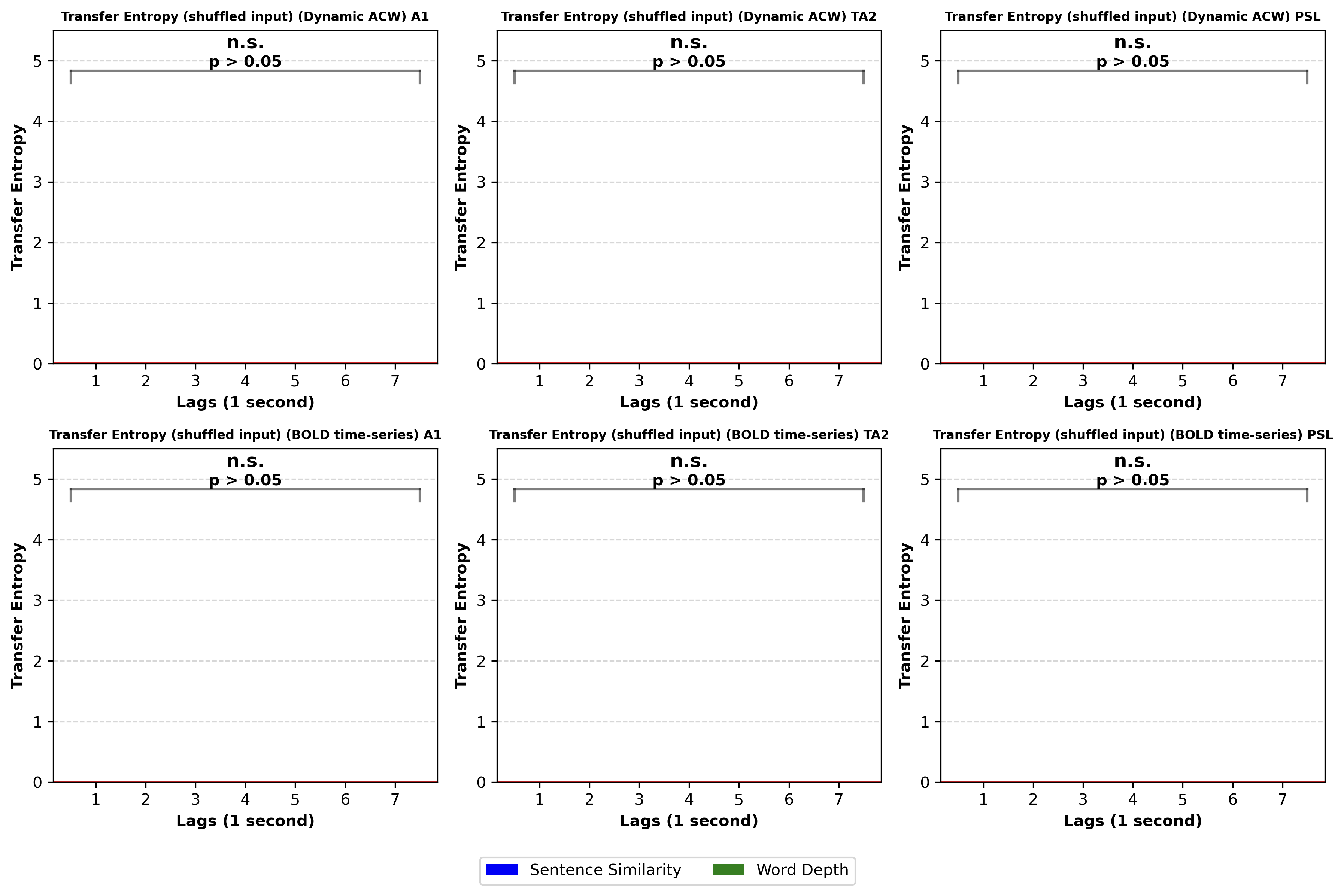

Supplement: Supplementary file 4 — Figure S4: Shuffling of Semantics disrupts information flow to the Brain. Information transfer (Transfer Entropy) from semantic input, shuffled using Markov Block Bootstrapping, to brain timescales during movie‐watching. (a) We computed Transfer Entropy in the input‐to‐brain direction between the shuffled semantic inputs' (sentence similarity and word depth) and the brain's dynamic ACW. (b) We also computed Transfer Entropy in the input‐to‐brain direction between the shuffled semantic inputs' (sentence similarity and word depth) time‐series and the brain's BOLD time‐series. (Statistics = Markov bootstrap procedure to estimate the statistical significance of Transfer Entropy; significance asterisks p < 0.05*, p < 0.01**, p < 0.001***). [file HBM-46-e70379-s005.tiff]
